# ‘From the core to beyond the margin’: a genomic picture of glioblastoma intratumor heterogeneity

**DOI:** 10.18632/oncotarget.3297

**Published:** 2015-04-16

**Authors:** Marc Aubry, Marie de Tayrac, Amandine Etcheverry, Anne Clavreul, Stéphan Saikali, Philippe Menei, Jean Mosser

**Affiliations:** ^1^ CNRS, UMR 6290, Institut de Génétique et Développement de Rennes (IGdR), Rennes F-35043, France; ^2^ Université Rennes1, UEB, UMS 3480 Biosit, Faculté de Médecine, Rennes F-35043, France; ^3^ Plate-forme Génomique Santé Biosit, Université Rennes1, Rennes F-35043, France; ^4^ CHU Rennes, Service de Génétique Moléculaire et Génomique, Rennes F-35033, France; ^5^ CHU Rennes, Service d’Anatomie et Cytologie Pathologiques, Rennes F-35033, France; ^6^ INSERM UMR-1066, Micro et Nano-Médecines Biomimétiques (MINT), Angers F-49933, France; ^7^ CHU Angers, Département de Neurochirurgie, Angers F-49933, France; ^8^ Service d’Anatomie Pathologique, Hôpital de l’Enfant-Jésus, Québec, Canada

**Keywords:** glioblastoma, intratumor heterogeneity, integrative functional genomics, invasion

## Abstract

Glioblastoma (GB) is a highly invasive primary brain tumor that almost systematically recurs despite aggressive therapies. One of the most challenging problems in therapy of GB is its extremely complex and heterogeneous molecular biology. To explore this heterogeneity, we performed a genome-wide integrative screening of three molecular levels: genome, transcriptome, and methylome. We analyzed tumor biopsies obtained by neuro-navigation in four distinct areas for 10 GB patients (necrotic zone, tumor zone, interface, and peripheral brain zone). We classified samples and deciphered a key genes signature of intratumor heterogeneity by Principal Component Analysis and Weighted Gene Co-expression Network Analysis. At the genome level, we identified common GB copy number alterations and but a strong interindividual molecular heterogeneity. Transcriptome analysis highlighted a pronounced intratumor architecture reflecting the surgical sampling plan of the study and identified gene modules associated with hallmarks of cancer. We provide a signature of key cancer-heterogeneity genes highly associated with the intratumor spatial gradient and show that it is enriched in genes with correlation between methylation and expression levels. Our study confirms that GBs are molecularly highly diverse and that a single tumor can harbor different transcriptional GB subtypes depending on its spatial architecture.

## INTRODUCTION

Glioblastoma (GB) is the most frequent and most aggressive malignant primary brain tumor [1, 2]. The current treatment strategy consists of surgery and concurrent adjuvant radiotherapy in combination with alkylating agents such as temozolomide, but GB prognosis remains extremely poor with a median survival of 12 to 15 months [3]. This uniformly poor prognosis of the disease is however associated with a notable variability at the histological level - as the ‘multiforme’ component of its former name implies - and by a striking molecular heterogeneity [4]. This heterogeneity has great significance for the general outcome of the malignancy as it intrinsically contributes to tumor aggressiveness and constitutes a clear issue for the design of effective therapies [5]. During recent years, many efforts were made to characterize GB molecular interpatient heterogeneity, and comprehensive profiling studies have identified underlying genomic and epigenomic aberrations that are associated with GB initiation and progression [6–10]. These molecular subclasses and signatures are however far from homogeneous and the question of their clinical relevance remains [11].

The clinical hallmarks of GB are its aggressive growth and inexorable recurrence despite multimodal therapy. Complete surgical resection of these infiltrative tumors is virtually impossible and GB almost systematically recurs after therapy, mainly at the margin of the resection cavity in the peritumoral brain zone [12, 13]. Deciphering GB intratumor heterogeneity at the molecular level is needed to understand this systematic recurrence. Intratumor heterogeneity, which refers to the presence of multiple, different cell subpopulations within a single tumor [14], contributes to tumor growth, progression and treatment failure [15]. In cancer, two complementary mechanisms have been proposed to explain this diversity in tumor cell populations: varying degrees of ‘stemness’ within cancer stem cells and clonal evolution [16–19]. In GB, the histopathologically observed coexistence of morphologically heterogeneous areas [20] has already been associated at the molecular level with different cells of origin [8], area-specific chromosome aberrations [21], mutations [22] and gene expression patterns [23, 24]. Comprehensive studies of GB intratumor heterogeneity are however rare and a better understanding of this heterogeneity will be essential to design effective therapies.

The *Grand Ouest Glioma Project: ‘from the core to beyond the margin’* (GOGP) is a translational program based on a multimodal analysis of GB areas [25–28]. This project aimed to provide insights on the origin of GB recurrence by characterizing GB intratumor heterogeneity. This intratumor heterogeneity was spatially defined from the core to the periphery: the necrosis, the tumor mass, the margin (interface between tumor and parenchyma, with decreasing tumor cells density), and isolated infiltrated cells in the normal parenchyma. While tumor mass and margin have been recently studied [24], necrotic and peripheral brain zones have been particularly neglected.

Here, we collected fragments in the four tumor zones for ten GB patients by means of computer-assisted neurosurgery. Our study presents an integrated genome-wide analysis of GB intratumor heterogeneity at three molecular levels: genome, transcriptome, and methylome. We show that GB intratumor heterogeneity is linked to genome variations and we highlight a strong modularity in the GB transcriptome. We then characterized a key cancer-heterogeneity genes signature linked to GB intratumor architecture. Our results confirm that tumor fragments from the same patient may be classified into different GB molecular subtypes.

## RESULTS

### Histological characterization of GB areas defined by MRI

The histology analysis of GB areas – necrotic zone (NZ), tumor zone (TZ), interface (I), and peripheral brain zone (PBZ) – by the central committee of neuropathologists is presented in Table 1. Seventy percent of NZ and TZ biopsy specimens presented the full histological features associated with a necrotic or a tumor zone. Biopsies in interface areas were more difficult to perform and mainly corresponded to macroscopically normal brain (3 patients), tumor tissue (2 patients) or infiltrated normal brain (1 patient). An infiltration by tumor cells (at least 10%) was observed in five PBZ.

**Table 1:** Histological characterization of GB areas defined by magnetic resonance imaging (MRI) Histological features are reported for each sample in terms of presence of necrosis, tumor tissue, infiltrating tumor cells and normal brain. +++: > 80%, ++ around 50%, + ≤ 30%, − < 10%. Gray denotes samples with low pre-analytic RNA quality controls (RIN).

Patient ID	MRI zone	necrosis	tumor tissue	infiltrating tumor cells	normal brain	Center
FT01	NZ	+++				Angers
	TZ	−	+++			
	I				+++	
	PBZ		+	+	+	
FT02	NZ	+++				Angers
	TZ	−		++	++	
	I		++	++		
	PBZ				+++	
FT03	NZ	+++				Angers
	TZ				+++	
	I			+++		
	PBZ		+		+++	
FT04	NZ	−	+++			Brest
	TZ	−	+++			
	I			+++		
	PBZ				+++	
FT05	NZ	+++				Brest
	TZ	−	+++			
	I				+++	
	PBZ				+++	
FT06	NZ	+++				Brest
	TZ	++	++			
	I	+	+	+		
	PBZ		+		+++	
FT07	NZ	+++				Rennes
	TZ	+	++		++	
	I		+++		−	
	PBZ	−	++		++	
FT08	NZ		+++			Rennes
	TZ		+++			
	I		+		++	
	PBZ		++		+	
FT09	NZ	+	+++			Rennes
	TZ	+	+++		−	
	I		+		+++	
	PBZ		−	+	+++	
FT13	NZ	+++				Tours
	TZ		−		+++	
	I		−		+++	
	PBZ		−		+++	

### Genomic profiling confirms common GB alterations

We profiled genome-wide DNA somatic copy number levels for 33 tumor fragments from the ten patients of the cohort. To investigate the global patterns of copy number alteration (CNA) within each patient, we took the union of CNAs that occurred in at least one of the sample biopsies (Supplementary File 1). We observed several frequent aberrations that have been reported in other GB cohorts, including loss/partial loss of chromosome 10 in all patients and the focal deletion of the *CDKN2A/B* locus in eight patients, as well as the frequently co-occurring deletion of *MTAP*. Polysomy of chromosome 7 was found in all patients. Focal high-level amplifications of *EGFR* were found in four patients. Moreover, we identified aberrations in several other known GB drivers, including focal amplification of *PDGFRA* (two cases), *SOX2* (two case), *MDM2* (one case), *MDM4* (one case), and *TERT* (two cases).

### Genome alterations and malignant clonal development

**Figure 1:**
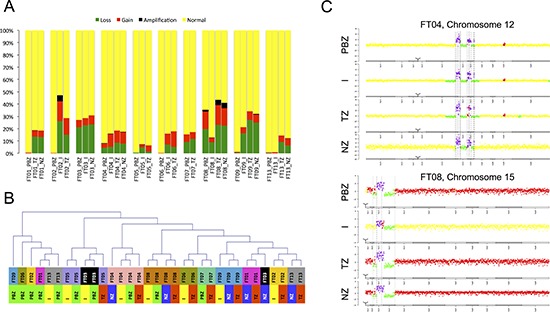
Genome profiling **A.** Copy Number Alterations (type and percentage) detected in each sample (yellow: normal, green: loss, red: gain, black: amplification). Samples are grouped by patient. **B.** Samples classification based on CNAs profiles. PBZ: peripheral brain zone, I: interface, TZ: tumor zone, NZ: necrotic zone. **C.** Examples of patient specific and atypical alterations.

For each biopsy, we classified CNAs as ‘normal’, ‘loss’, ‘gain’ or ‘amplification’ (Figure 1A). Samples with less than 1% of altered profile were considered potential normal brain zones or slightly infiltrated areas. For the other samples, we observed a strong heterogeneity in terms of percentage of altered profiles. This heterogeneity was mostly inter-individual as samples from the same patient showed a relatively stable percentage of altered profiles. FT02 and FT08 tumors were the most altered, with more than 30% of altered profiles and a high proportion of amplifications, whereas all FT05 samples presented only a small fraction of alterations (less than 10%). Samples classification based on CNAs profiles confirmed the distinction between altered and non-altered samples. Samples harboring more than 1% of altered profiles were grouped in one cluster separated from potential normal brain zones or slightly infiltrated areas (Figure 1B). This cluster highlighted very similar alteration profiles within samples originating from the same patient, particularly for FT04 and FT08. These similarities were associated with some patient specific and atypical alterations, on chromosome 12 for FT04 and on chromosome 15 for FT08 (Figure 1C).

Following the work of Sottoriva et al. on cancer evolutionary dynamics [24], we interrogated copy number alterations to explore intratumor temporal spectre of evolution based on our sampling plan. We inspected copy number alterations manually and with the TuMult algorithm [29]. We confirm that copy number alterations targeting chromosomes 7 and 10 are among the earliest events in GB tumor evolution, and particularly loss of *CDKN2A/B* and *EGFR* amplification. Other alterations such as amplifications of *PDGFRA*, *MDM2* and *MDM4* were also identified as important events occurring at different steps of tumor expansion depending on the patient.

### Transcriptome profiling confirms strong intratumor heterogeneity

**Figure 2:**
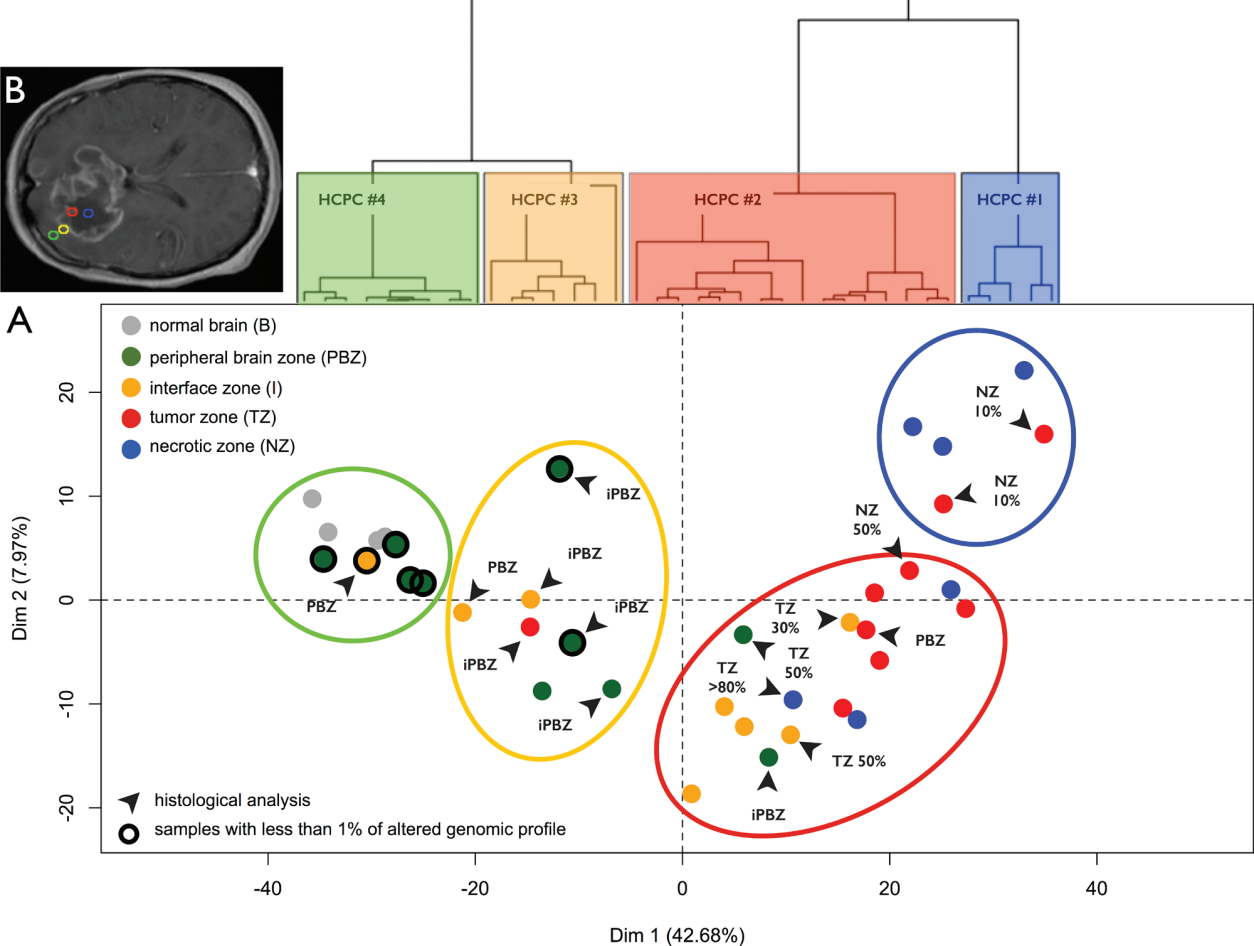
Transcriptome profiling **A.** Principal Component Analysis (PCA) performed on the expression data for 41000 probes without *a priori* selection. Dots represent samples and are colored according to the neuro-navigation sampling: green (PBZ: peripheral brain zone), yellow (I: interface zone), red (TZ: tumor zone), and blue (NZ: necrotic zone). Gray dots represent normal brain reference samples. Dendrogram of the hierarchical clustering based on principal components (HCPC) is represented above the Individual factor map. HCPC clusters are represented on the factorial plan by colored ellipses reflecting the sampling plan of the study ‘from the core of the tumor to beyond the margin’: HCPC #4 (blue), HCPC #3 (red), HCPC #2 (yellow), and HCPC #1 (green). Samples with unaltered array-CGH profile are circled in black. Black arrows designate samples with non-concordant histological analysis (PBZ: non-infiltrated parenchyma, iPBZ: infiltrated parenchyma, I: interface, TZ x%: presence of a corresponding percentage of tumor cells, and NZ x%: presence of a corresponding percentage of necrotic cells). **B.** Areas for biopsy in the four GB zones defined on preoperative MRI: necrotic zone (blue), tumor zone (red), interface (yellow), and peripheral brain zone (green).

PCA and HCPC performed on the whole microarray dataset highlighted zone-specific profiles with pronounced intra-tumor architecture: from the core to beyond the margin (Figure 2A). This classification reflected the surgical sampling plan of the study (Figure 2B), highlighting that the transcriptome heterogeneity was much more important within tumors than between patients. This classification was confronted with histological examinations performed by the central committee of neuropathologists and copy number alteration analyses. Clusters were enriched as follow: tumor and necrotic biopsies (HCPC #1), tumor and interface biopsies (HCPC #2), infiltrated peripheral brain zone (HCPC #3), and, reference control brain biopsies with peripheral brain zone biopsies (HCPC #4). All samples in the latter cluster were identified as non-tumorous zones by neuropathologists and presented less than 1% of altered profile.

### Glioblastoma subtype depends on tumor area

**Figure 3:**
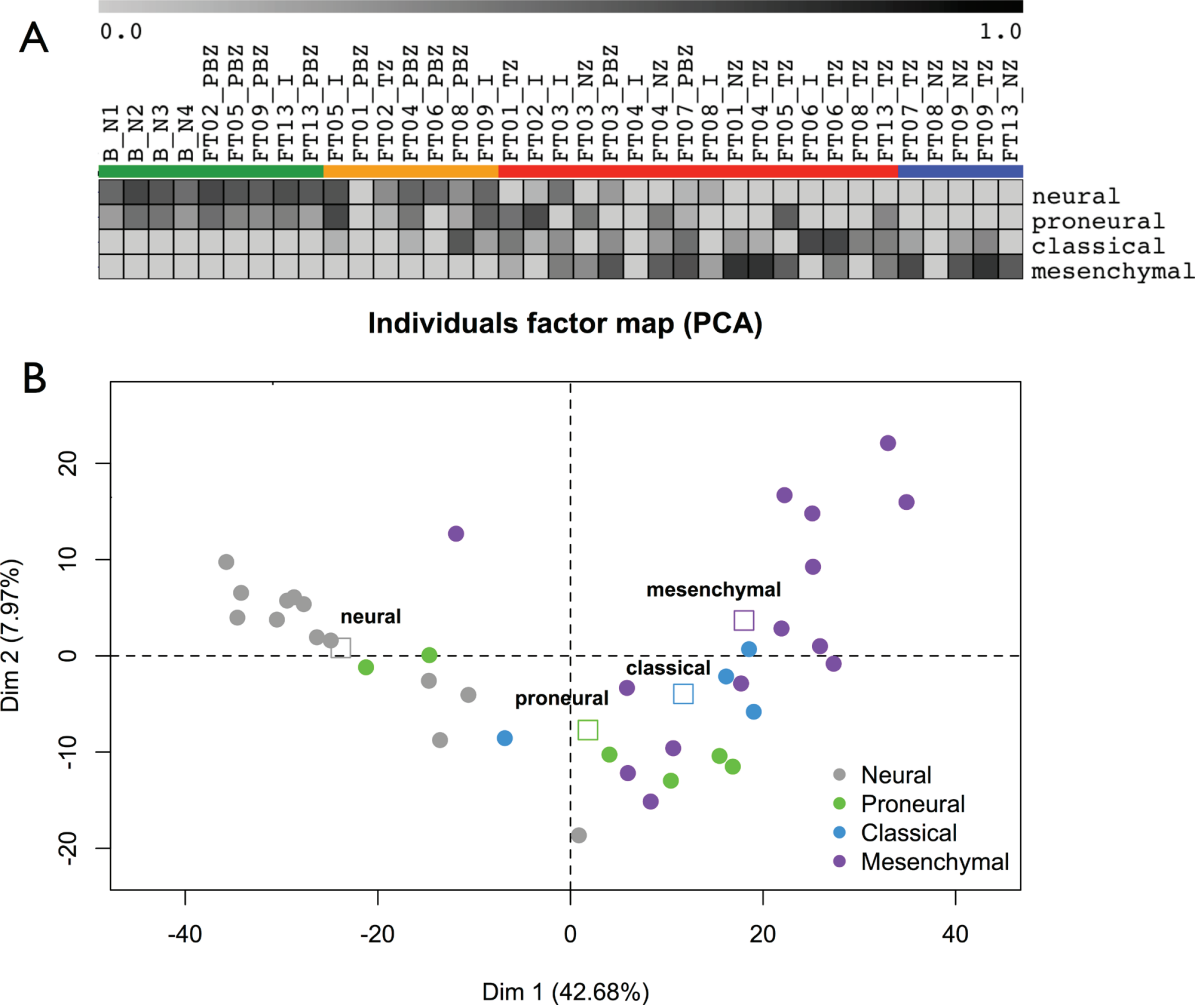
GB subtypes **A.** Samples GB subtypes according to the Verhaak signature. Gene Set Enrichment Analysis enrichment scores of each samples are reported as a gray-based color gradient. **B.** Samples GB subtypes and zone-specific profiles determined by PCA. Samples are colored according to their GB subtype. Squares: barycenter of GB subtypes.

We assigned each biopsy to one of four subtypes: *Proneural*, *Neural*, *Classical*, and *Mesenchymal* using the Verhaak classifier [10], which is based on an 840-gene signature. Samples from peripheral brain zone (PBZ) and interface (I) biopsies showed the highest correlations with the *Neural* or *Proneural* subtypes. In contrast, tumor (TZ) and necrotic (NZ) biopsies showed the highest correlations with the *Mesenchymal* and *Classical* subtypes (Figure 3A). All samples in HCPC #4 were classified as *Neural*. In the other HCPC clusters, we found that in 9 of 10 cases, biopsies from the same patient were classified into at least two different subtypes. Only FT07 was classified as *Mesenchymal* on both PBZ and TZ biopsies. FT02 was classified as *Neural* and *Proneural* indicating a strong *Neural* component in this tumor. In the other cases, we observed mainly the combination [(*Neural* or *Proneural*) and *Mesenchymal*] (6 cases), but also the [(*Neural* or *Proneural*) and *Classical*] combination (1 case). FT08 presented strong tumor heterogeneity with two *Mesenchymal* and two *Classical* biopsies (Supplementary File 2). Taken as a whole, the Verhaak GB classes were highly associated with the zone-specific profiles, as determined by the PCA performed on the whole transcriptome dataset (ANOVA, *p* = 9.10-9) (Figure 3B). This highlighted that the definition of GB subtype based on gene expression was related to the biopsy zone.

### Transcriptome architecture of intratumor heterogeneity

**Figure 4:**
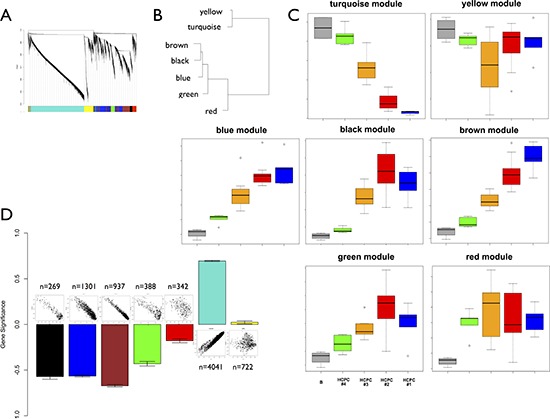
Weighted gene co-expression network analysis **A.** Cluster dendrogram and co-expression modules. **B.** Clustering of module eigengenes. **C.** Module eigengenes expression across the sampling plan. Boxplots are colored according to HCPC classification. The gray boxplot represents reference control brain samples (*n* = 4). The green boxplot includes HCPC1 samples minus reference control brain samples. **D.** Gene significance (mean and standard error) for each module. Above each bar is a scatter plot of gene significance (GS) versus module membership (MM). GS is based on *p*-values from the ANOVA performed between HCPC clusters (BH corrected *p*-values). Number of probes in each module is also reported.

We performed weighted gene co-expression network analysis to map genes and pathways onto the intra-tumor heterogeneity highlighted by PCA. We restricted this analysis to the 8000 most informative probes (see methods). It identified seven co-expression modules hierarchically organized into two distinct groups (Figure 4A and Figure 4B). The fist group gathered 60% of the genes and was composed of only two modules (turquoise and yellow) whereas the second group was composed of five modules. The turquoise and yellow modules gathered the downregulated genes from the periphery to the center of the tumor (PBZ to TZ/NZ biopsies). The five other modules (black, blue, brown, green and red) gathered the probes upregulated from the periphery to the center of the tumor (Figure 4C). We identified modules related to the intratumor heterogeneity by computing a gene significance measure for each probe. This measure identified four significant modules: black, blue, brown and turquoise (Figure 4D). These modules are very coherent as they showed strong correlations between gene significance and module membership. Functional enrichments were performed to assess the biological significance of each module (Table 2 and Supplementary File 3). This analysis pointed out a clear distinction between the turquoise/yellow modules and the other modules. The turquoise and yellow modules were characterized by a strong composition of genes involved in the nervous system development and function. The five other modules were mainly characterized by genes involved in cellular growth and proliferation (3 modules), cellular development (3 modules), cellular movement (3 modules), and cell cycle (2 modules). Modules were highly coherent as core modules displayed functional enrichments similar to those of entire modules (Supplementary File 4). Based on the Verhaak signature, we found that the brown module was enriched with *Mesenchymal* genes (chi-squared test, *p* < 1e-16) and that the turquoise module was enriched with *Proneural* (*p* = 0.002) and *Neural* (*p* = 0.02) genes.

**Table 2:** Enriched functional categories best associated with co-expression modules For each module are reported molecular and cellular functions, networks and upstream regulators significantly associated in the Ingenuity Pathways Analysis database.

Module	Molecular and Cellular Functions	Top Networks	Top Upstream Regulators
turquoise	Molecular Transport Cell-To-Cell Signaling and Interaction Nervous System Development and Function Cell Morphology	Neurological Disease, Developmental Disorder, Renal and Urological Disease Infectious Disease, Cell Signaling, Vitamin and Mineral Metabolism Cell-To-Cell Signaling and Interaction, Nervous System Development and Function, Cell Morphology Cell-To-Cell Signaling and Interaction, Molecular Transport, Small Molecule Biochemistry	SBDS miR-122-5p CASR SOAT1 FSH
black	Cell Cycle Cellular Assembly and Organization DNA Replication, Recombination, and Repair Cellular Growth and Proliferation Cellular Development	Cell Cycle, DNA Replication, Recombination, and Repair, Cancer Cell Cycle, DNA Replication, Recombination, and Repair, Cellular Assembly and Organization Cell Cycle, Cellular Assembly and Organization, DNA Replication, Recombination, and Repair Cell Cycle, Cancer, Reproductive System Disease Cell Cycle, Cell Death and Survival, DNA Replication, Recombination, and Repair	E2F4 CCND1 CDK4 ERBB2 CDKN1A
blue	Cell Cycle Gene Expression RNA Post-Transcriptional Modification Cellular Movement Connective Tissue Development and Function Embryonic Development	RNA Post-Transcriptional Modification, Molecular Transport, RNA Trafficking Embryonic Development, Organismal Development, Tissue Development Hair and Skin Development and Function, Organ Morphology, Cell Morphology Infectious Disease, Cellular Development, Embryonic Development Cellular Assembly and Organization, Nervous System Development and Function, Cell Morphology	MYC mir-15 FANCC LYL1 E2F4
brown	Cellular Movement Cell Death and Survival Cellular Growth and Proliferation Cell-To-Cell Signaling and Interaction Cellular Development	Cancer, Organ Development, Respiratory Disease Cancer, Gastrointestinal Disease, Cell Death and Survival Infectious Disease, Cell Death and Survival, Antimicrobial Response Cellular Movement, Hematological System Development and Function, Immune Cell Trafficking Cellular Movement, Cardiovascular System Development and Function, Organismal Development	TNF IFNG MAPK1 TGFB1 IFNA2
green	Cellular Development Cellular Growth and Proliferation Cellular Movement Hematological System Development and Function Immune Cell Trafficking	Infectious Disease, Cellular Function and Maintenance, Cell-To-Cell Signaling and Interaction Cellular Movement, Hematological System Development and Function, Immune Cell Trafficking Cellular Function and Maintenance, Cellular Movement, Immune Cell Trafficking Humoral Immune Response, Protein Synthesis, Cellular Development Cell-To-Cell Signaling and Interaction, Cellular Growth and Prolif., Hematological System Dev. & Function	IFNG RFX5 TNF IFN alpha/beta SMC3

### Gene expression signature of intratumor heterogeneity is marked by correlation with methylation levels

**Figure 5:**
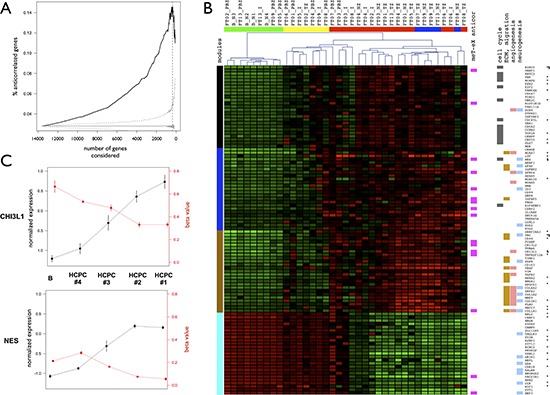
Anti-correlated genes and master genes signature **A.** Percentages of anti-correlated genes between expression and methylation levels. Genes are ranked according to their importance in terms of contribution to the PCA data structure and percentages are calculated for subsets of genes ranging from the 100 most important genes to the whole dataset (x-axis). Black: observed percentage. Gray: significativity of the percentage of anti-correlated genes estimated by bootstrapping (*n* = 1000). Median (solid line) and CI 99% (dotted) are presented. Vertical dashed line: maximum percentage obtained for 370 genes. **B.** Master genes signature. Heatmap and samples hierarchical clustering for transcriptome data. Samples are colored by HCPC cluster (see Figure 2a for details). Genes functional insights are represented by cluster colors. Left: coexpression modules. Right: anti-correlation between methylation and expression levels, statistically enriched Gene Ontology functional categories. Validated differential expression are also reported (*: transcriptome data, tumor versus normal; $: RT-qPCR data, tumor zone versus peripheral brain zone). **C.** Normalized expression levels and methylation beta-values across HCPC clusters for *CHI3L1* (brown module) and *NES* (blue module). Means +/− standard errors for each HCPC cluster with normal brain control samples set apart (see Figure 4C for details).

PCA performed on the methylome data (27578 CpG sites, 23 samples) showed the presence of particular intratumor architecture as observed at the transcriptome level (Supplementary File 5). There were an estimated 3% (382 genes) of genes showing expression-methylation correlations (*r* < −0.6) in the whole dataset. To assess the importance of epigenetics mechanisms in the definition of intratumor heterogeneity, we analyzed the proportion of anti-correlated genes with respect to their contribution to the transcriptome PCA data structure. This proportion was compared to random samplings (*n* = 1000) of the genes. The percentage of anti-correlated genes significantly increased with the gene contribution to the PCA data structure. The highest percentage of anti-correlated genes (15%, 54 genes) was reached for the top 370 genes (Figure 5A).

### A signature of key cancer-heterogeneity genes highly associated with the intratumor spatial gradient

We focused on the difference between infiltrated brain zone and macroscopically normal brain (HCPC #3 and HCPC #4, respectively). We selected the 100 most informative genes in terms of (i) importance in the PCA data structure, (ii) differential expression, and (iii) module membership (25 genes per significant module). Among these genes we found well-known GB genes such as: *VEGF*, *CD44*, *GFAP*, *EZH2*, *CHI3L1*, *NES*, and *IGFBP2*. Hierarchical clustering based on this selection conserved the inner structure of the data identified by PCA and the biological meaning of the coexpression modules (Figure 5B and Supplementary File 6). As for the co-expression analysis, this suggested that central genes drove the functional enrichment of each module. Particularly, we observed an enrichment of genes associated with cell cycle in the black module (13 genes, 87%) and genes associated with ECM and migration in the brown module (15 genes, 79%). We found 19 genes directly associated with neurogenesis (7 in the turquoise module). The angiogenesis process was also significantly enriched in this 100-gene signature (14 genes). Thirty-four genes were also identified as strongly differentially expressed between GB and normal brain in our previous study [30] (Figure 5B). Sixteen genes presented an anti-correlation between methylation and expression levels, and particularly in the blue and brown modules. As an illustration, Figure 5C presents expression and methylation levels for *CHI3L1* (brown module) and *NES* (blue module).

### External validations of the key cancer-heterogeneity signature

We performed external validations of the key cancer-heterogeneity signature with two independent studies of GB intratumor heterogeneity [24, 31]: the Sottoriva et al. microarray study of 52 samples (10 patients) and the Gill et al. RNA-Seq study of 92 samples (27 patients). In both studies, hierarchical clustering of the samples based on the key cancer-heterogeneity signature showed a similar gene expression pattern (Supplementary File 7 and Supplementary File 8). It allowed a clear distinction between tumor biopsies and margin/macroscopically normal brain biopsies. In the Sottoriva et al. study, tumor biopsies clustered into two groups: one enriched in *Proneural* subtypes and the other in *Classical*/*Mesenchymal* subtypes. Most of the time, different tumor samples from the same patient were classified into these different subgroups. In both studies, our key cancer-heterogeneity signature showed that the *Neural* subtype was clearly associated with biopsies performed in the margin/non-enhancing tumor region.

## DISCUSSION

The genomic profiling of the GB samples included in our study confirmed the presence of common glioblastoma alterations reported in previous studies [4]: loss/partial loss of chromosome 10, polysomy of chromosome 7, focal deletion of the CDKN2A/B and focal high-level amplifications of EGFR. Altered samples presented a strong heterogeneity, which was mostly interindividual. Core and interface biopsies presented a higher amount of genomic alterations compared to biopsies performed beyond the tumor margins. A study of intratumor temporal dynamics confirmed that copy number alterations targeting chromosomes 7 and 10 are among the earliest events in GB tumor evolution. It was however not possible to identify specific genomic copy number alterations characterizing the peripheral brain zone.

At the transcriptome level, we observed that the molecular heterogeneity was much more important within tumors than between patients. PCA-based analysis highlighted zone-specific profiles with pronounced intratumor architecture reflecting the surgical sampling plan of the study: ‘from the core to beyond the margin’. Our findings also support the implicit notion of coexpression network analysis that genes with similar expression behavior are related biologically. Indeed, the gene modules identified and their functional enrichments clearly evidenced modularity in the GB transcriptome, a feature already evidenced by Bredel and colleagues [32]. These modules could be associated with several hallmarks of cancer [33], highlighting a strong level of self-organization in the GB transcriptome: sustaining proliferative signaling and evading growth suppressors (black module), activating invasion and metastasis, inducing angiogenesis and resisting cell death (brown module), avoiding immune destruction and promoting inflammation (blue and green modules), promoting genome instability and mutation (as exemplified by CNA analysis). Co-expression modules were not specifically associated with a biopsy area. One reason could be a lack of power due to the small sample size of the study. This could have been improved by increasing the number of peripheral brain or interface zones, which is difficult in the context of human studies.

Our results confirmed that GB tumors are molecularly diverse but not randomly so. Indeed, we were able to identify a hundred key genes which expression was highly correlated with the surgical sampling plan: from the core to beyond the margin. In accordance with other molecular descriptions of GB intratumor heterogeneity [24, 31], our results confirmed that the core is an area of high proliferation and inflammation and that tumor margins are infiltrated brain areas which transcriptome is influenced by the presence of oligodendrocytes and neurons. This signature includes genes previously associated with GB cells infiltrative behavior. Chitinase 3-like 1 (*CHI3L1*/*YKL-40*, brown module) is a mesenchymal marker that promotes tumor angiogenesis and malignancy [34]. It is a marker of prognosis and disease status in high-grade gliomas [6, 35–37]. Nestin (*NES*, blue module) is a stem cell marker that regulates the migration, invasion and growth of human gliomas [38]. In GB, its expression increases with tumor grade in both tumor cells and endothelial cells [39]. Nestin has also been proposed as a useful marker for examining the infiltration of malignant astrocytic tumors cells into the surrounding tissue [40].

Methylome and transcriptome studies captured a similar intratumor architecture depicting tumor spatial heterogeneity with 3% of the genes displaying an inverse correlation between promoter methylation and expression levels [7]. When focusing on the key genes signature the percentage of anti-correlated genes was five times higher than expected, indicating that their abnormal expression might be linked to cis-acting epimutations in their promoter. We hypothesized that these targeted epimutations could be a consequence of alterations affecting regulators of epigenetic gene silencing [41]. We found two such regulators coexpressed in the black module: *UHRF1* and *EZH2*. It has been shown that they synergistically promote the inactivation of tumor suppressor genes in colorectal cancer [42]. This result is consistent with the fact that the disruption of the epigenetic machineries may precede the classical preliminary transforming events (mutations and genomic instability) and provoke aberrant gene expression patterns that give rise to all typical cancer characteristics [41].

Recent large-scale genomic analyses have revealed patterns of molecular changes within tumor subclasses that harbor distinct underlying biology, clinical prognosis, and pathogenic routes [10, 11, 43–46]. There remains no consensus on the number of clinically meaningful transcriptional GB subtypes but virtually all studies to date identify a key distinction between tumor subtypes with features that are described as *Proneural* and *Mesenchymal* [11, 47]. This lack of consensus has been mostly attributed to technical variation (*e.g*., platform, batch effect, sample size) and biological noise (variability among tumors) [47, 48]. It is however important to note that these molecular subclasses were defined on data obtained on a unique tumor sample per patient, from which percentage and type of tumor cells could vary. As a corollary, many tumors do not fit neatly into one group or another, as they may be composed of multiple and/or molecularly distinct subpopulations. Our results clearly evidenced that a single tumor can harbor different transcriptional GB subtypes depending on its spatial architecture and that the *Neural* subtype is associated with interface and peripheral brain zones. We confirmed that the *Mesenchymal*, *Classical*, and *Proneural* GB subtypes are robustly associated with GB tumor tissue but that the *Neural* subtype should be taken with caution because it is clearly associated with the tumor margin or non-enhancing region.

## PATIENTS AND METHODS

### Patients and tissue samples

Twenty-six patients were enrolled between 2006 and 2011 in the Grand Ouest Glioma Project (GOGP), a prospective and multicentric study. All patients were diagnosed for a *de novo* GB in one of the Neurosurgical Departments of the University Hospitals of Angers, Brest, Poitiers, Rennes or Tours (France). Approval by the local ethic committee of the entire project (CPP Ouest II; Angers, France) was obtained prior to the initiation of the study. Ten patients from the GOGP were included in the present study. Diagnosis of primary GB [20] was confirmed by a central committee of neuropathologists for all patients. Areas for biopsies were defined on preoperative T1 gadolinium-enhanced 3D MRI in four different zones of the GB tumors. The sampling plan of these four zones was designed ‘from the core of the tumor to beyond the margin’: necrotic zone (NZ), tumor zone (TZ), interface (I), and peripheral brain zone (PBZ). Stereotaxic biopsies were performed in the operating theater, by computer-assisted neurosurgery (BrainLAB^®^, La Défense, France). PBZ biopsies were all performed about 2 cm outside the contrast enhancement, in the T2 hypersignal on MRI. In total, 40 biopsies (4 per patient) were immediately snap-frozen in liquid nitrogen and kept at −80°C until used. Histological analysis was realized for each specimen on formalin-fixed paraffin-embedded sections. Four brain parenchyma biopsies obtained from patients who underwent cortectomy for epilepsy were used as reference samples. Blood samples were collected for each patient and used to extract reference DNA for CGH array analysis.

### DNA and RNA isolation

DNA was extracted with the NucleoSpin Tissue Kit (Macherey Nagel) according to the manufacturer's instructions. The quality of DNA biopsies was assessed by electrophoresis in a 1% agarose gel. Total RNA was isolated with the NucleoSpin RNAII Kit (Macherey-Nagel). RNA integrity (RNA Integrity Number ≥ 8) was confirmed with an Agilent 2100 Bioanalyzer (Agilent Technologies). Pre-analytic RNA quality controls based on RNA integrity identified 7 low-quality samples. For these samples, genomic analysis was not performed (4 necrotic zones: FT02_NZ, FT05_NZ, FT06_NZ and FT07_NZ; 1 tumoral zone: FT03_TZ; 2 interfaces: FT01_I and FT07_I).

### Array CGH profiling and data analysis

Array CGH profiling was performed with the Human CGH 4x44K Microarray Kit (Agilent Technologies) according to the manufacturer's recommendations. For each patient, each biopsy DNA and matching patient constitutional DNA were pooled and hybridized together to avoid false positives due to individual copy number variation. Genomic profiles were computed and compared using waviCGH software [49]. Normalization was performed by subtracting the weighted median from the log-ratios for each array. Duplicated probes were merged by the median. Segmentation was performed using the *HaarSeg* method [50]. Each segment was assigned a probability of being lost (–1), normal (0), gained (+1) or amplified (+2) using the *CGHcall* method [51]. Minimal common regions (MCRs) were identified using a permutation method taking into account the frequency of calls in each sample and chromosome to calculate statistically significant MCRs. We used the TuMult algorithm [29] to reconstruct the lineage of tumor samples from the same patient, together with the sequence of chromosomal events occurring during tumorigenesis. Copy number arrays have been deposited in the Gene Expression Omnibus repository under the accession number GSEXXXXX.

### Gene expression profiling and normalization

Gene expression profiling was carried out with the Agilent Whole Human Genome 4x44K Microarray Kit (Agilent Technologies). Total RNA was extracted, labelled and hybridized according to the kit manufacturer's recommendations. Raw intensity data were log2-transformed and normalized (intra-array and inter-array scaling) using the R *limma* package. Expression arrays have been deposited in the Gene Expression Omnibus repository under the accession number GSEXXXXX.

### DNA methylation profiling

DNA methylation profiling was performed with the Infinium HumanMethylation27 beadchip (Illumina Inc.). DNA from GBM samples and control brains were bisulfite-modified, using the EZ DNA methylation kit (Zymo Research) and hybridized according to the manufacturer's instructions. For each interrogated CpG site, methylation status is calculated by dividing the signal from the methylated probe (M) by the sum of signals for both methylated and unmethylated (U) probes (*Genome Studio 2008.1*, Illumina Inc.): β-value = Max(M, 0)/ [Max(M, 0) + Max(U, 0) + 100]. This β-value provides a continuous and quantitative measurement of DNA methylation, ranging from 0 (completely unmethylated) to 1 (completely methylated). Missing values were imputed by nearest neighbors averaging (*impute* R package). All samples were G-CIMP negative [9]. All samples were *MGMT* unmethylated [52] except FT06_TZ and FT06_I. Methylation arrays have been deposited in the Gene Expression Omnibus repository under the accession number GSEXXXXX.

### Anti-correlated genes

Correlations between transcriptome and methylome data were computed on a gene basis for all the genes with available data on the two molecular levels (*n* = 13612). Anti-correlated genes were defined as genes presenting a negative correlation between expression and methylation levels (Pearson correlation coefficient less than –0.6).

### Sample classification and gene selections

We used Principal Component Analysis (PCA) and Hierarchical Clustering on Principal Components (HCPC) to classify samples and select genes. PCA is a linear dimensionality reduction technique (factorial analysis) that seeks to identify a small number of components that capture most of the relevant structure in a dataset. HCPC is an agglomerative hierarchical classification method based on the principal components of the factorial analysis. PCA and HCPC were performed on transcriptome data with the *FactoMiner* R package and default parameters. Following the rationale of the experimental design of the study, we cut the hierarchical tree in order to obtain four clusters. We also used PCA to identify informative genes as done by Paschou et al. [53] in the field of Populational Genetics. They have developed a method to select informative markers related to the data structure revealed by PCA. This selection algorithm first determines the number of significant principal components (and thus the number of informative eigenMarkers) in the data. Subsequently, a score is assigned to each marker, with higher scores corresponding to markers that correlate well with all informative eigenMarkers. The markers are ranked according to this score and the top scoring markers are selected for further analyses. When computing the scores we used the correlation with the first Principal Component only. The gene expression dataset was reduced to the top 8000 genes for subsequent analyses.

### Differential expression analysis

A one-way Welch's anova was performed to compare the mean gene expression levels between HCPC clusters and to identify genes up- or downregulated in at least one cluster. Adjusted *p*-values were calculated by controlling for the FDR with the Benjamini & Hochberg (BH) procedure. Absolute fold-changes (FC) between two conditions were also computed. Genes were considered significantly differentially expressed between 2 conditions for an adjusted *p*-value lower than 0.001 and an absolute FC greater than 2.

### Co-expression network analysis

We constructed a signed weighted gene co-expression network on the basis of the expression data of the most informative genes. Gene co-expression networks are increasingly used to explore the system-level functionality of genes. The network construction is conceptually straightforward: nodes represent genes that are connected if the corresponding genes are significantly co-expressed across appropriately chosen tissue samples. In a weighted gene co-expression network, a connection weight is assigned to each gene pair. This weight reflects the co-expression measure between the two genes (correlation) and is based on the network spatial relationship between these genes (adjacency) [54]. Pearson correlation coefficients were calculated across all samples for all possible pairs of the variable genes. The correlation matrix was raised to the power 12, thus producing a weighted network. This weighted network was transformed into a network of topological overlap (TO) — an advanced co-expression measure that considers not only the correlation of two genes with each other, but also the extent of their shared correlations across the weighted network. Genes were hierarchically clustered on the basis of their TO. Modules were identified on the dendrogram using the Dynamic Tree Cut algorithm [55]. Each gene's connectivity was determined within its module of residence (intramodular connectivity) by summing up the TOs of the gene with all the other genes in the module. A module membership (MM) measure was defined for all input genes (irrespective of their original module membership) as the correlation of its expression profile with the module eigengene of a given module. The module membership measure being highly related to intramodular connectivity, it was used to identify hub genes (highly connected intramodular genes) displaying characteristic expression profiles for their module of residence. Gene significance (GS) measures the association (correlation) between a gene and external data. Here, GS was based on the adjusted *p*-values from the analysis of variance performed between HCPC clusters. All network analyses were performed using the *WGCNA* R package with default parameters except for the minModuleSize that was set to 175. Functional analyses were generated through the use of IPA (Ingenuity® Systems, www.ingenuity.com) and GOMiner [56]. For each co-expression module, functional enrichments were tested for the whole module and for a ‘core module’ defined as the upper quartile of the genes ranked on decreasing module membership.

### Real-time quantitative PCR (Q-PCR)

Q-PCR reactions were done with the 7900HT Fast Real-Time PCR System using the SYBR TMGreen PCR Master Mix (Applied Biosystems). *GAPDH* (glyceraldehyde-3-phosphate dehydrogenase) and *TBP* (TATA box binding protein) RNAs were chosen as internal controls. Calibration was performed with FirstChoice Human Brain Reference Total RNA (Ambion). The relative amounts of the gene transcripts were determined using the delta-delta-Ct method. Primers are provided in Supplementary File 9.

## SUPPLEMENTARY FIGURES










