# Correction: From the core to beyond the margin: a genomic picture of glioblastoma intratumor heterogeneity

**DOI:** 10.18632/oncotarget.12550

**Published:** 2016-10-10

**Authors:** Marc Aubry, Marie de Tayrac, Amandine Etcheverry, Anne Clavreul, Stéphan Saikali, Philippe Menei, Jean Mosser

**Present**: Due to an error made in manuscript preparation, the microarrray data was ascribed to the wrong repository institutions.

**Correct**: The correct repository assignments are provided below. The authors sincerely apologize for this oversight.

Original article: Oncotarget. 2015; 6(14):12094-109. doi: 10.18632/oncotarget.3297.

The repository information should be amended as follows:

‘Copy number arrays have been deposited in the ArrayExpress repository under the accession number E-MTAB-5098′.

‘Expression arrays have been deposited in the ArrayExpress repository under the accession number E-MTAB-5092′

‘Methylation arrays have been deposited in the ArrayExpress repository under the accession number E-MTAB-5109′

