# Supplementary material for: ‘From the core to beyond the margin’: a genomic picture of glioblastoma intratumor heterogeneity
Source: Oncotarget. 2015 Apr 16;6(14):12094–109. doi: 10.18632/oncotarget.3297 (PMC4494925; doi:10.18632/oncotarget.3297)
Supplement: Supplementary file 1 [file oncotarget-06-12094-s001.pdf]

SUPPLEMENTARY FILES

A

	necro.	tumor	inter.	normal
NZ	+++			
TZ	-	+++		
I				+++
PBZ		+	+	+

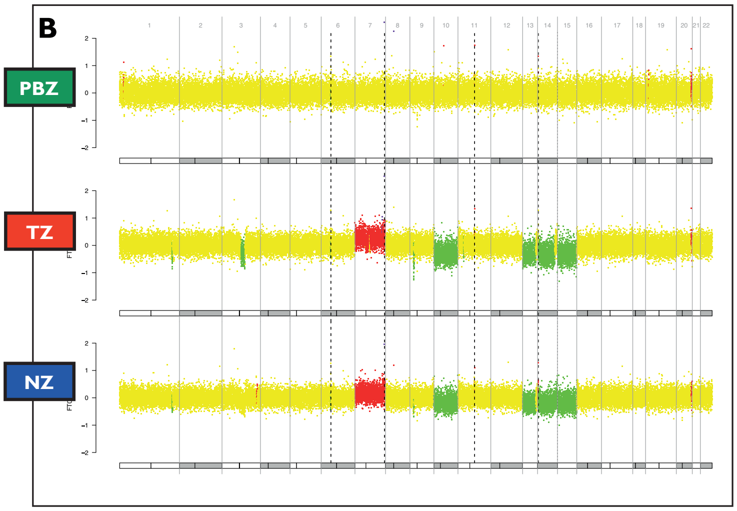

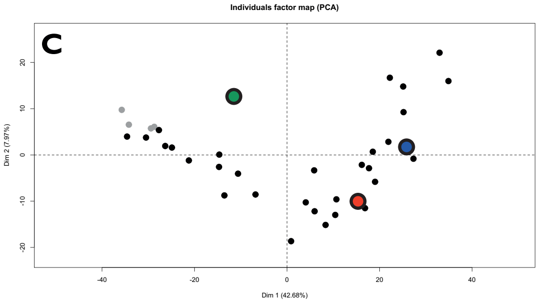

FT01 / GB-3

peripheral brain zone (PBZ) +++ >80%
interface zone (I) ++ around 50%
tumor zone (TZ) + ≤30%
necrotic zone (NZ) - <10%

A - Histological analysis
B - Copy number alteration profile
C - Expression profile

KEY

A

	necro.	tumor	inter.	normal
NZ	+++			
TZ	-		++	++
I		++	++	
PBZ				+++

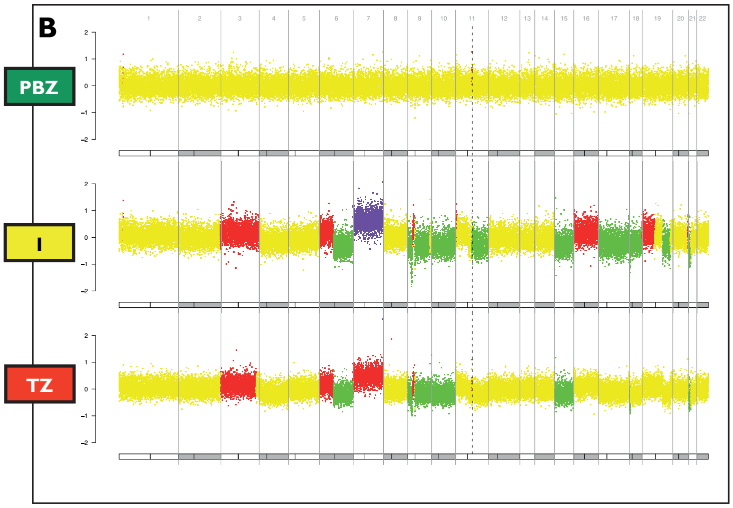

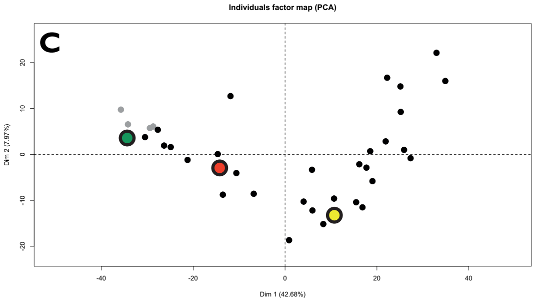

FT02 / GB-9

peripheral brain zone (PBZ) +++ >80%
interface zone (I) ++ around 50%
tumor zone (TZ) + ≤30%
necrotic zone (NZ) - <10%

A - Histological analysis
B - Copy number alteration profile
C - Expression profile

KEY

(Continued)

A

	necro.	tumor	inter.	normal
NZ	+++			
TZ				+++
I			+++	
PBZ		+		+++

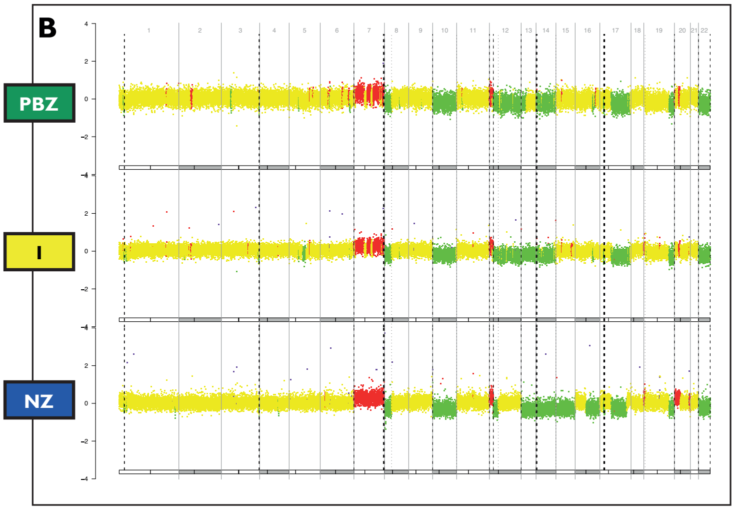

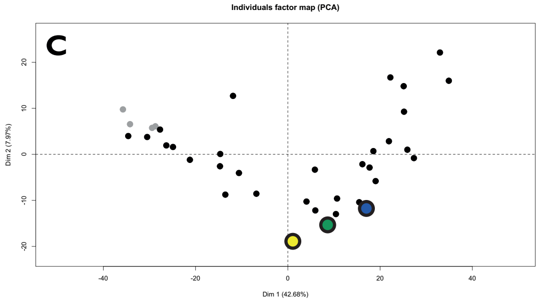

FT03 / GB-10

peripheral brain zone (PBZ)
interface zone (I)
tumor zone (TZ)
necrotic zone (NZ)

+++ >80%
++ around 50%
+ ≤30%
- <10%

A - Histological analysis
B - Copy number alteration profile
C - Expression profile

KEY

A

	necro.	tumor	inter.	normal
NZ	-	+++		
TZ	-	+++		
I			+++	
PBZ				+++

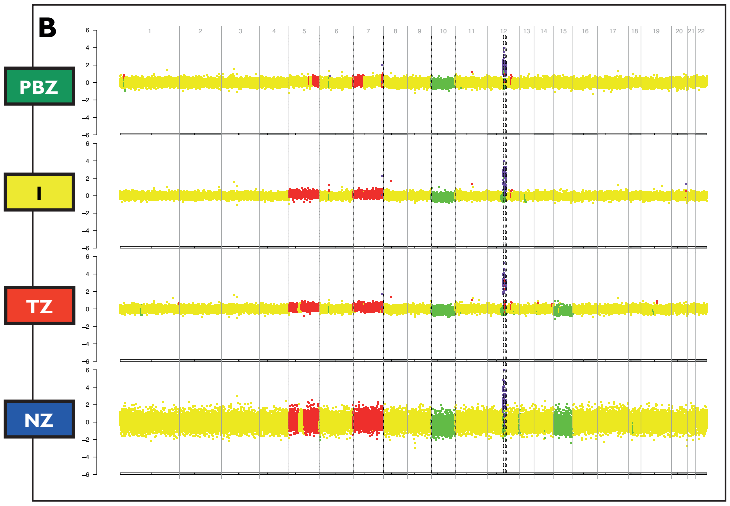

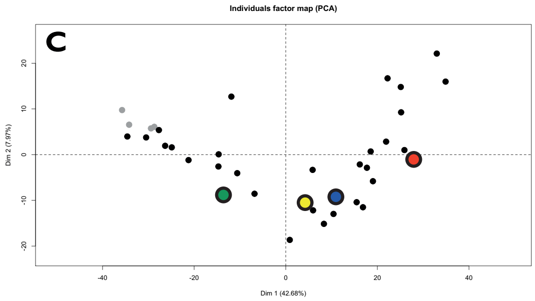

FT04 / GB-15

peripheral brain zone (PBZ)
interface zone (I)
tumor zone (TZ)
necrotic zone (NZ)

+++ >80%
++ around 50%
+ ≤30%
- <10%

A - Histological analysis
B - Copy number alteration profile
C - Expression profile

KEY

(Continued)

A

	necro.	tumor	inter.	normal
NZ	+++			
TZ	-	+++		
I				+++
PBZ				+++

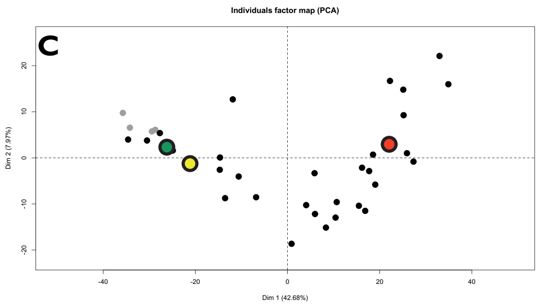

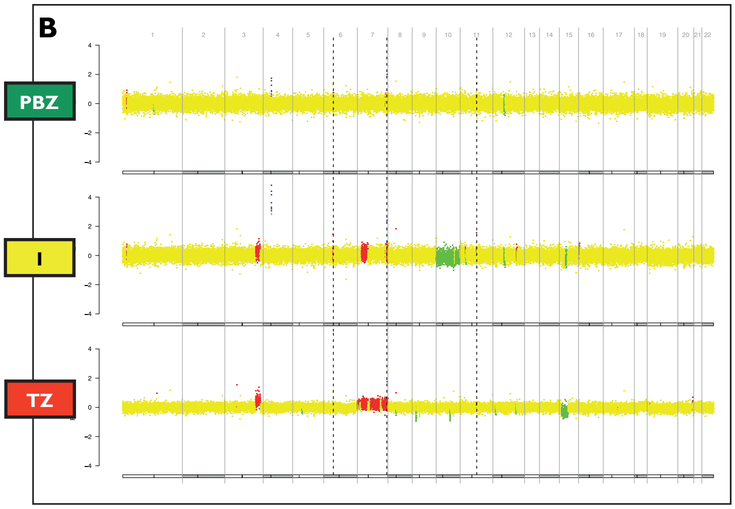

FT05 / GB-16

peripheral brain zone (PBZ) +++ >80%
interface zone (I) ++ around 50%
tumor zone (TZ) + ≤30%
necrotic zone (NZ) - <10%

A - Histological analysis
B - Copy number alteration profile
C - Expression profile

KEY

A

	necro.	tumor	inter.	normal
NZ	+++			
TZ	++	++		
I	+	+	+	
PBZ		+		+++

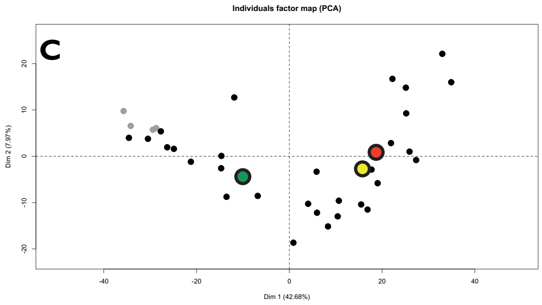

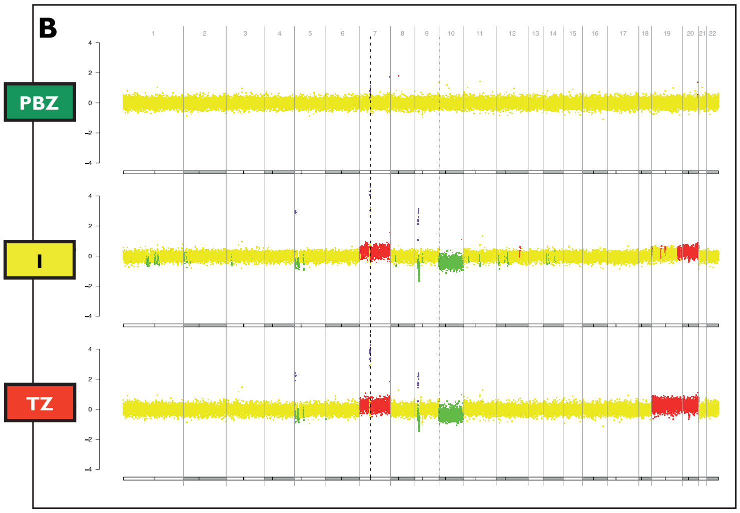

FT06 / GB-17

peripheral brain zone (PBZ) +++ >80%
interface zone (I) ++ around 50%
tumor zone (TZ) + ≤30%
necrotic zone (NZ) - <10%

A - Histological analysis
B - Copy number alteration profile
C - Expression profile

KEY

(*Continued*)

A

	necro.	tumor	inter.	normal
NZ	+++			
TZ	+	++		++
I		+++		-
PBZ	-	++		++

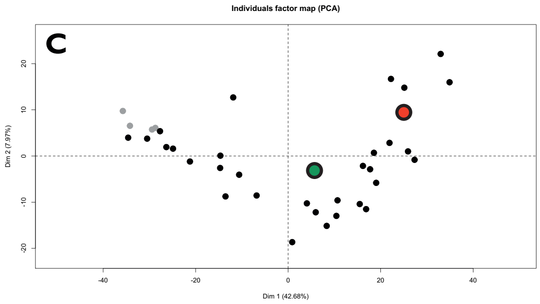

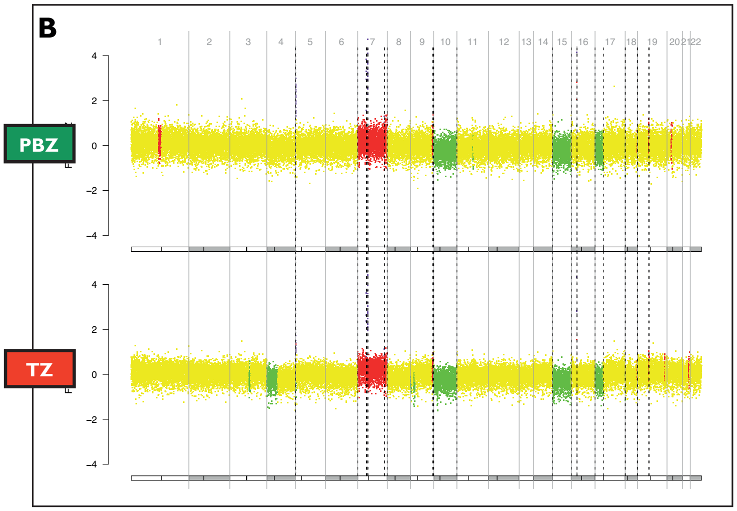

FT07 / GB-35

KEY

- peripheral brain zone (PBZ)
- interface zone (I)
- tumor zone (TZ)
- necrotic zone (NZ)

+++ >80%
++ around 50%
+ ≤30%
- <10%

A - Histological analysis
B - Copy number alteration profile
C - Expression profile

A

	necro.	tumor	inter.	normal
NZ		+++		
TZ		+++		
I		+		++
PBZ		++		+

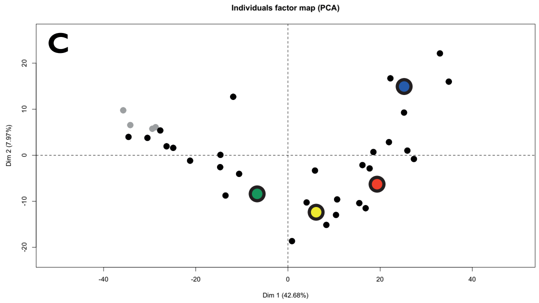

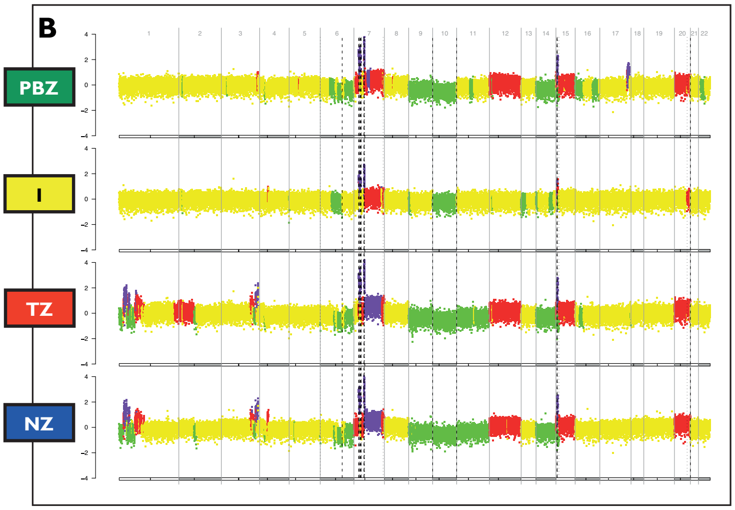

FT08 / GB-36

KEY

- peripheral brain zone (PBZ)
- interface zone (I)
- tumor zone (TZ)
- necrotic zone (NZ)

+++ >80%
++ around 50%
+ ≤30%
- <10%

A - Histological analysis
B - Copy number alteration profile
C - Expression profile

(*Continued*)

A

	necro.	tumor	inter.	normal
NZ	+	+++		
TZ	+	+++		-
I		+		+++
PBZ		-	+	+++

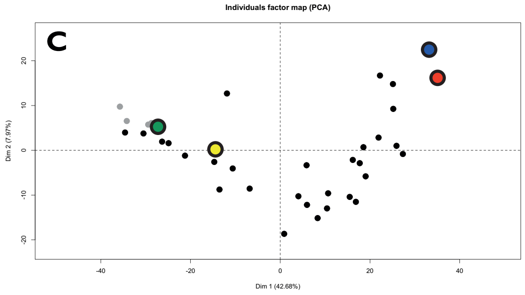

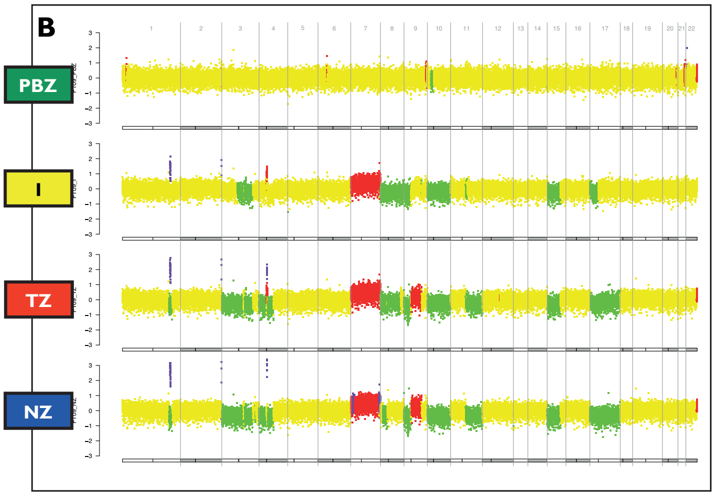

FT09 / GB-25

- peripheral brain zone (PBZ)
- interface zone (I)
- tumor zone (TZ)
- necrotic zone (NZ)

+++ >80%
++ around 50%
+ ≤30%
- <10%

A - Histological analysis
B - Copy number alteration profile
C - Expression profile

KEY

A

	necro.	tumor	inter.	normal
NZ	+++			
TZ		-		+++
I		-		+++
PBZ		-		+++

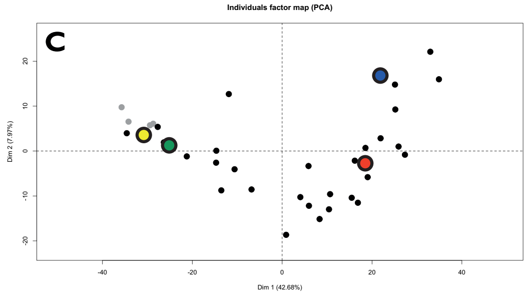

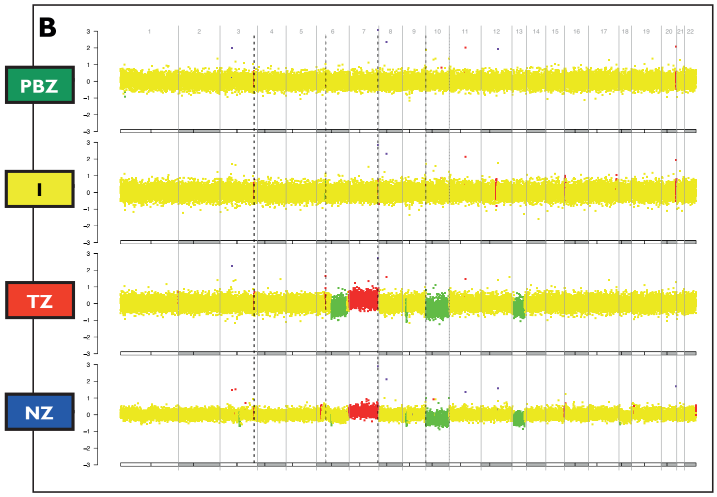

FT13 / GB-26

- peripheral brain zone (PBZ)
- interface zone (I)
- tumor zone (TZ)
- necrotic zone (NZ)

+++ >80%
++ around 50%
+ ≤30%
- <10%

A - Histological analysis
B - Copy number alteration profile
C - Expression profile

KEY

Supplementary Files S1: Histological, genome and transcriptome profiles of patient biopsies. Samples are colored according to the neuro-navigation sampling: green (PBZ: peripheral brain zone), yellow (I: interface zone), red (TZ: tumor zone), and blue (NZ: necrotic zone). All these sample-based analyses were compiled to produce Figure 2A. **A.** Histological analysis: percentage of necrotic cells, tumor cells, infiltrating cells and normal cells in each sample. **B.** Genome profile: copy number alterations (yellow: normal, green: loss, red: gain, violet: amplification). **C.** Transcriptome profile: samples position on the PCA first factorial plan.

Supplementary File S2: Samples annotations. Sample id, patient ids, biopsy zone, hospital center, HCPC cluster and GB molecular subtype based on Verhaak signature.

Supplementary File S3: Co-expression modules functional annotations (GOMiner). Functional enrichments are reported for each coexpression module: Gene Ontology (GO) id and term, total number of genes associated with GO category, number of module-genes associated with GO category, enrichment score, *p*-value (log10) of the enrichment score.

Supplementary File S4: Enriched functional categories best associated with co-expression core modules. For each core module are reported molecular and cellular functions, networks and upstream regulators significantly associated in the Ingenuity Pathways Analysis database.

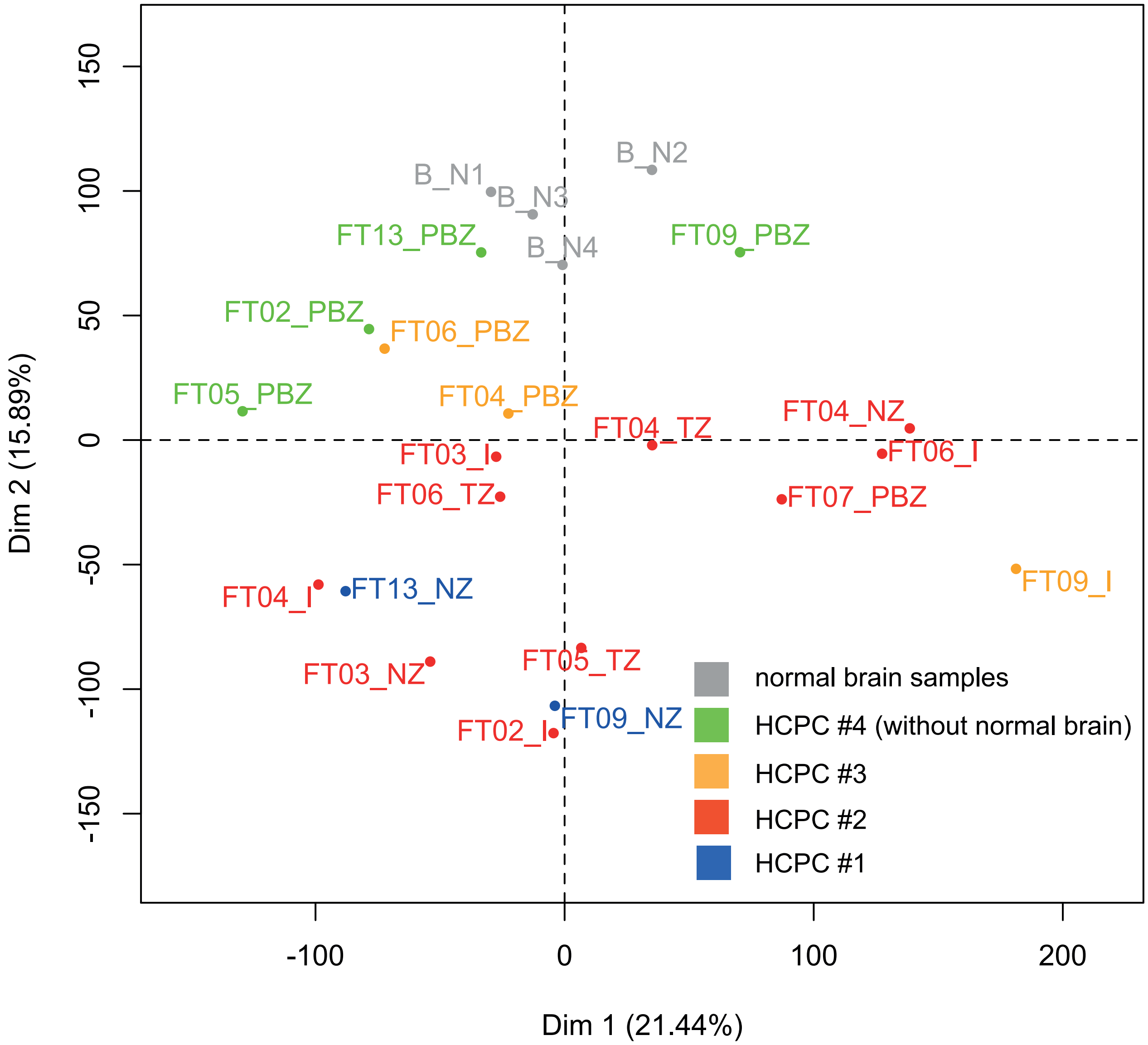

Supplementary File S5: Intratumor architecture revealed by PCA performed on methylome data (27578 CpG sites) for 23 samples. Gray dots represent normal brain reference samples. All other samples are colored by HCPC cluster. Gradient 'from the core to beyond the margin' can be observed on PC2. It shows the presence of an intra-tumor architecture as observed at the transcriptome level.

Molecular functions

	p-value
Cellular Movement	4,19E-12 - 8,84E-03
Cellular Growth and Proliferation	1,56E-10 - 8,79E-03
Cell Death and Survival	8,93E-10 - 9,34E-03
Cellular Development	1,13E-08 - 9,57E-03
Cell Cycle	2,69E-07 - 7,46E-03

Upstream regulators

	p-value of overlap
TGFB1	1,51E-21
TNF	3,76E-12
ERBB2	3,27E-10
HDAC6	6,48E-08
PPARG	8,59E-08

Canonical pathways

	p-value
Hepatic Fibrosis / Hepatic Stellate Cell Activation	1,54E-06
Glioma Invasiveness Signaling	3,62E-04
Pancreatic Adenocarcinoma Signaling	4,2E-04
Intrinsic Prothrombin Activation Pathway	5,97E-04
Atherosclerosis Signaling	7,39E-04

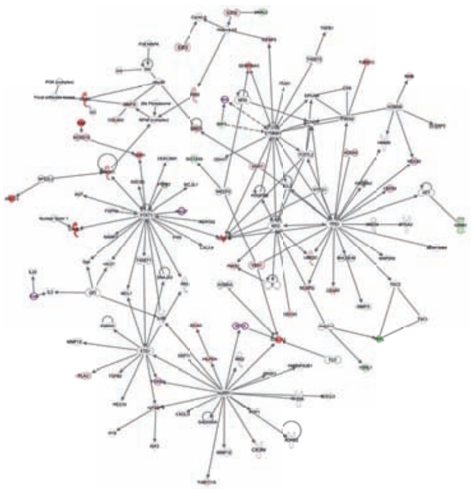

Diseases & bio. functions

Category	Diseases or Functions Annotation	p-Value	# Molecules
Cancer	glioma cancer	1,10E-20	22
Neurological Disease	glioma cancer	1,10E-20	22
Cancer	glioblastoma cancer	9,68E-20	21
Neurological Disease	glioblastoma cancer	9,68E-20	21
Cancer	brain cancer	1,44E-19	23
Neurological Disease	brain cancer	1,44E-19	23
Cancer	head and neck tumor	7,08E-18	33
Cancer	astrocytoma	2,15E-16	24
Neurological Disease	astrocytoma	2,15E-16	24
Cancer	head and neck cancer	6,75E-16	30
Cancer	central nervous system tumor	5,47E-15	25
Neurological Disease	central nervous system tumor	5,47E-15	25
Cellular Movement	cell movement	4,19E-12	32
Cancer	pelvic cancer	7,69E-11	33
Cancer	genital tumor	8,11E-11	28
Organismal Injury and Abnormalities	genital tumor	8,11E-11	28
Reproductive System Disease	genital tumor	8,11E-11	28
Cellular Growth and Proliferation	proliferation of cells	1,56E-10	45
Cellular Movement	migration of cells	1,74E-10	28
Cell Death and Survival	apoptosis	8,93E-10	35
Cell Death and Survival	cell death	4,07E-09	39
Cancer	Cancer	4,60E-09	81
Cellular Growth and Proliferation	proliferation of tumor cell lines	1,13E-08	30
Cellular Development	proliferation of tumor cell lines	1,13E-08	30
Cellular Movement	cell movement of tumor cell lines	1,50E-08	21
Cell Death and Survival	necrosis	1,91E-08	34
Cell Death and Survival	cell death of tumor cell lines	2,04E-08	30
Cancer	malignant neoplasm of abdomen	2,06E-08	60
Cell Death and Survival	apoptosis of tumor cell lines	2,06E-08	26
Cancer	breast or ovarian cancer	2,60E-08	32
Cancer	renal cancer	5,02E-08	13
Organismal Injury and Abnormalities	renal cancer	5,02E-08	13

Molecules in Network	Focus Molecu	Top Diseases and Functions
26s Proteasome, ADCYAP1, Akt, ↑**ANXA2**, ↑**ANXA5**, ↑**BIRC5**, CALD1, ↑**CCNB2**, ↑**CD44**, ↑**CD99**, ↑**CD163**, ↑**CHI3L1**, ↑**COL1A1**, ↑**COL4A2**, COL6A1, COL6A2, Cyclin A, DLL4, ↑**E2F2**, EDNRB, EFEMP1, ↑**EIF4EBP1**, ERK, ERK1/2, estrogen receptor, ↑**EZH2**, FBLN2, Focal adhesion kinase, GC, ↑**GFAP**, Gm-csf, HAS3, Histone h3, ↑**HMOX1**, ↑**HOXD10**, Hsp90, ↑**ID3**, ↑**IGFBP2**, ↑**IGFBP5**, Igm, IL12 (complex), ↑**IL1RAP**, IRS, Jnk, ↑**LTF**, ↓**MAL2**, MAP2K1/2, ↑**MDK**, ↑**MMP9**, NFkB (complex), Nuclear factor 1, P38 MAPK, ↑**PBK**, PDGF BB, PI3K (complex), ↑**PLAU**, ↑**PTPRZ1**, Ras, ↑**SERPINA3**, ↑**SFRP4**, ↑**SOD2**, ↑**SOX4**, TCF, ↑**TIMP1**, ↑**TNC**, ↑**TOP2A**, tyrosine kinase, Vegf, ↑**VEGFA**, ↑**VIM**	36	Cellular Movement, Cancer, Neurological Disease
BCL2, BHLHE40, CCND1, CD9, ↑**CDC45**, ↑**CDCA7**, CDH11, ↑**CENPF**, ↑**CEP55**, ↑**COL1A2**, ↑**COL3A1**, CTNNB1, ↑**DLGAP5**, EPCAM, FBN1, FLI1, ↑**FMOD**, ↓**GRIN1**, HMMR, ↑**HOXA5**, HTT, ↑**IGFBP5**, ITGA1, ITGA2, KDM5A, KDM5B, let-7a-5p (and other miRNAs w/seed GAGGUAG), MAP2K6, MECP2, MMP3, MST1R, MYC, ↑**NCAPG**, ↑**NDC80**, ↓**NEFL**, ↑**NES**, NFIX, ↑**NMB**, NOX4, ↓**PACSIN1**, PCDH11X/PCDH11Y, PDGFRB, PECAM1, PSEN2, PTTG1, ↑**SERPINA3**, SERPINB5, ↓**SLC12A5**, SLC5A8, ↑**SOX4**, T, TCF7L2, TGFB1, ↑**TGFBI**, ↑**TNFRSF12A**, TP53, TSC1, TSC2, ↑**TUBA1C**, TWIST2, TYMP, ↑**UBE2C**, ↑**UHRF1**, ↑**VEGFA**, ↓**VSNL1**, ↓**WIF1**, Wnt, WNT5A, ↑**YBX1**, ZNF217	29	Cellular Movement, Cell Death and Survival, Cellular Growth and Proliferation
↑**ABCC3**, ADIPOQ, ADORA2B, ADRB2, AGT, BCL2L1, ↓**CCK**, CD86, ↑**CDCA2**, CEACAM1, ↑**CHI3L1**, ↑**COL1A2**, Creb, CXCL3, CXCL5, CXCL9, CXCR4, DNAJA3, ETS1, ↑**FAM111A**, FGF1, FGFR2, FN1, ↑**FOXD1**, FZD8, GADD45A, GDF15, GFI1, H19, ↑**HILPDA**, ↑**HMOX1**, HNRNPA2B1, Hsp27, IGF2, ↑**IGF2BP3**, ↑**IGFBP2**, IL2, IL22, IL27, IL17A, IL6R, IRS2, ITGB2, MCL1, mir-21, MMP12, MMP13, NAMPT, ↓**NEFM**, NFE2L2, ↓**NRGN**, NUPR1, OSM, ↑**PDPN**, PEG10, ↑**PLAU**, PSEN1, ↓**PTPRT**, PXN, RFX5, SOCS3, ↑**SRPX**, SRSF1, STAT3, ↓**SYT1**, TGFB2, ↑**TIMP1**, TNF, TWIST1, ↑**VEGFA**	20	Cell Death and Survival, Cell-To-Cell Signaling and Interaction, Organismal Injury and Abnormalities

Supplementary File S6: Master genes functional annotations (Ingenuity Pathways Analysis). Enrichment *p*-values associated with molecular functions, upstream regulators, canonical pathways and diseases & biological functions. Molecules and top diseases and functions are reported for the top three networks associated with the master genes signature. Master genes appear in bold. Red(Green) arrows indicate overexpression(underexpression) in the tumor. Merged network of the top three networks associated with the master genes signature is also presented (solid line: direct association, dashed line: indirect association, red/green: differential expression along the sampling plan).

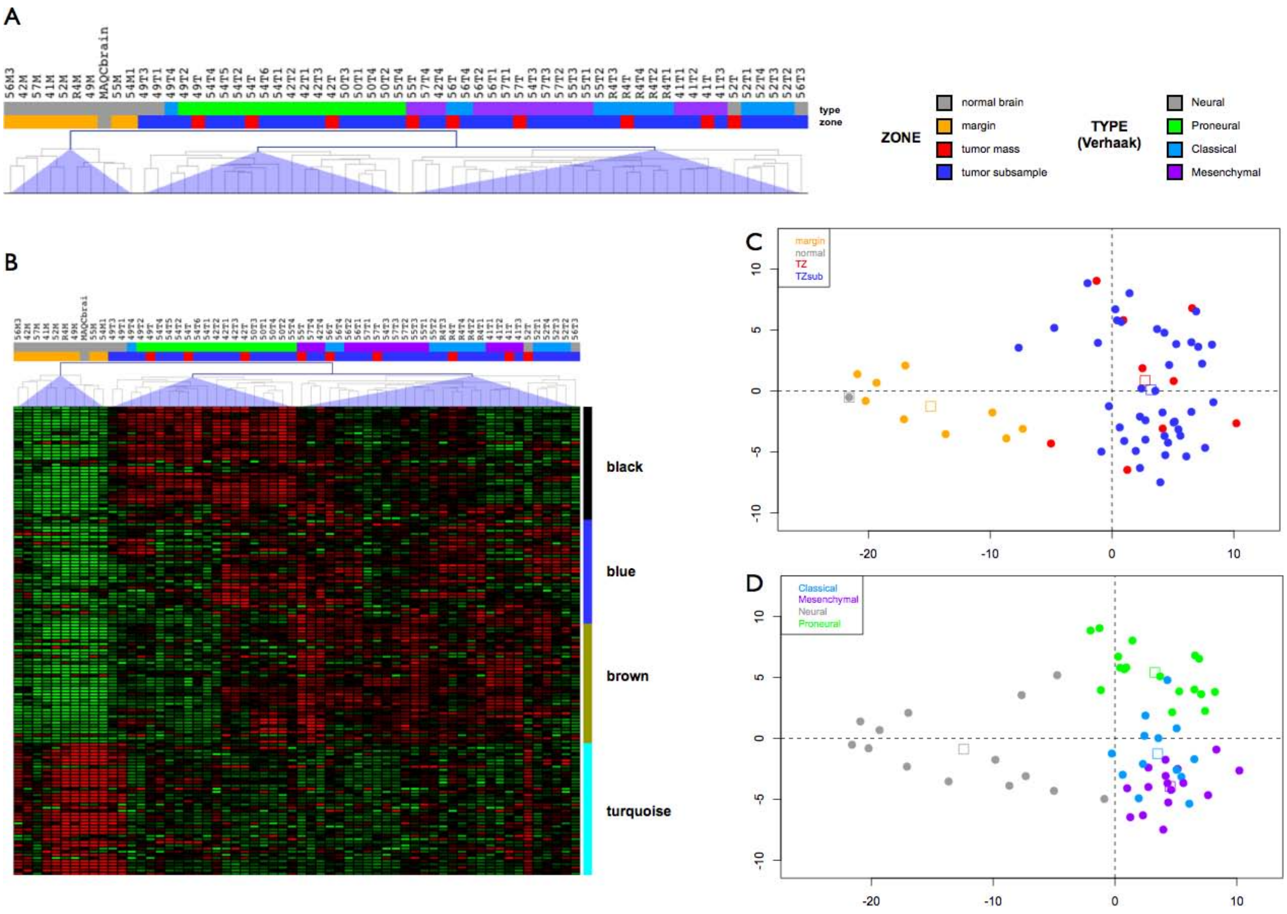

Supplementary File S7: External validation of the key cancer-heterogeneity signature. Validation performed on Sottoriva et al. GB dataset (PNAS, 2013). **A.** Samples hierarchical clustering for transcriptome data. Samples are colored (i) by GB molecular subtype based on Verhaak signature, and (ii) by biopsy zone (see color legend for details). **B**. Heatmap and coexpression modules. **C.** PCA analysis based on transcriptome data. Samples are colored by biopsy zone. **D.** PCA analysis based on transcriptome data. Samples are colored by GB subtype. These panels highlight the same intratumor architecture as observed with the data generated in our study.

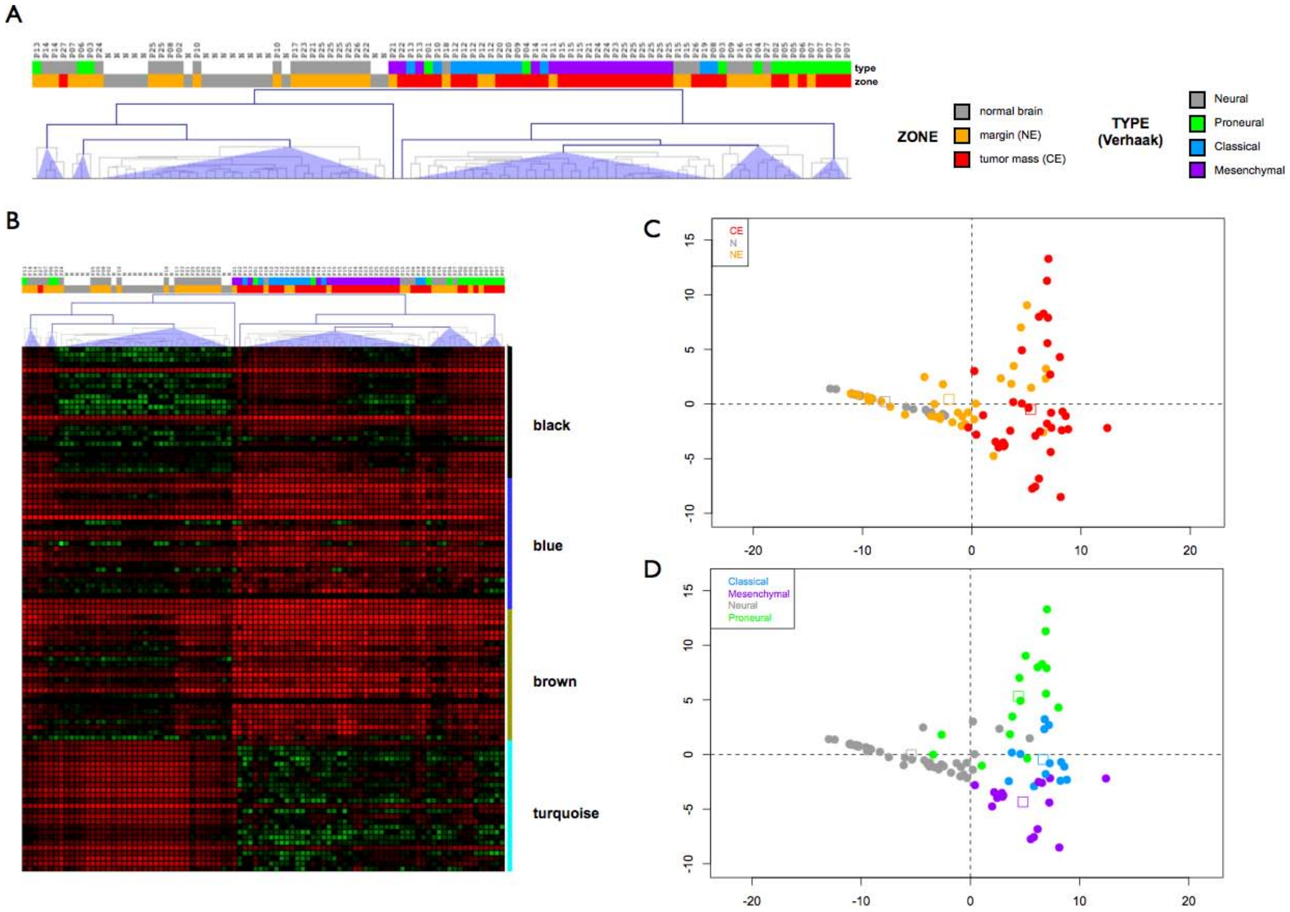

Supplementary File S8: External validation of the key cancer-heterogeneity signature. Validation performed on Gill et al. GB dataset (PNAS, 2014). **A.** Samples hierarchical clustering for transcriptome data. Samples are colored (i) by GB molecular subtype based on Verhaak signature, and (ii) by biopsy zone (see color legend for details). **B**. Heatmap and coexpression modules. **C.** PCA analysis based on transcriptome data. Samples are colored by biopsy zone. **D.** PCA analysis based on transcriptome data. Samples are colored by GB subtype. These panels highlight the same intratumor architecture as observed with the data generated in our study.

Supplementary File S9: Primers used for RT-qPCR validation. The expression levels of 4 master genes (*BIRC5*, *NES*, *TNC* and *CHI3L1*) were assessed by RT-qPCR to validate their differential expression along the sampling plan (tumor zone versus peripheral brain zone).
