# Supplementary material for: ‘From the core to beyond the margin’: a genomic picture of glioblastoma intratumor heterogeneity
Source: Oncotarget. 2015 Apr 16;6(14):12094–109. doi: 10.18632/oncotarget.3297 (PMC4494925; doi:10.18632/oncotarget.3297)

**Additional File 3**

Black module


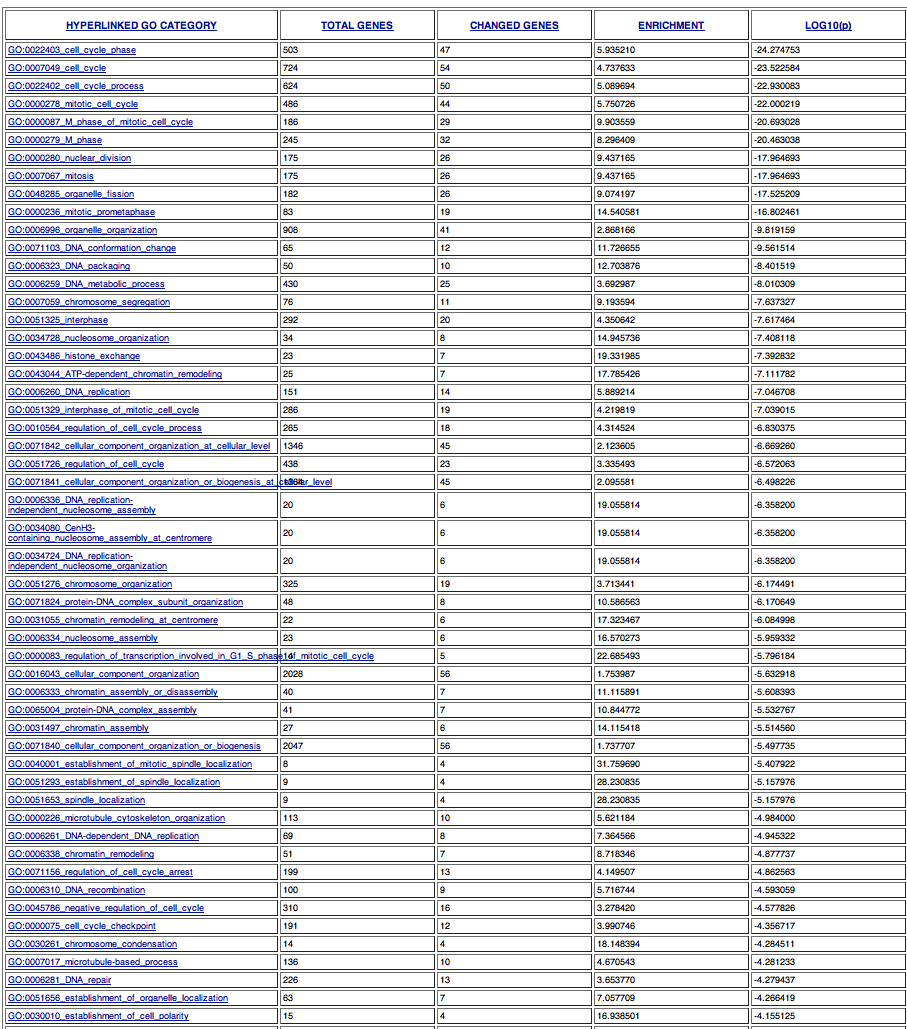


Blue module


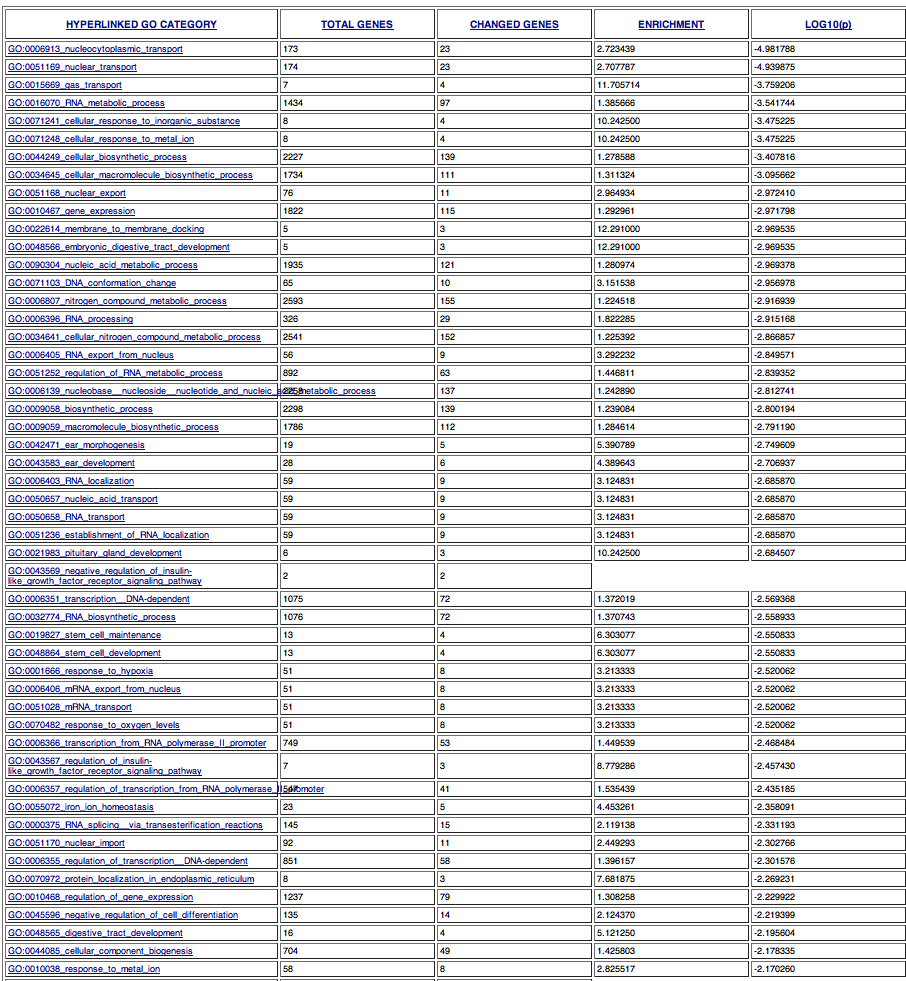


Brown module


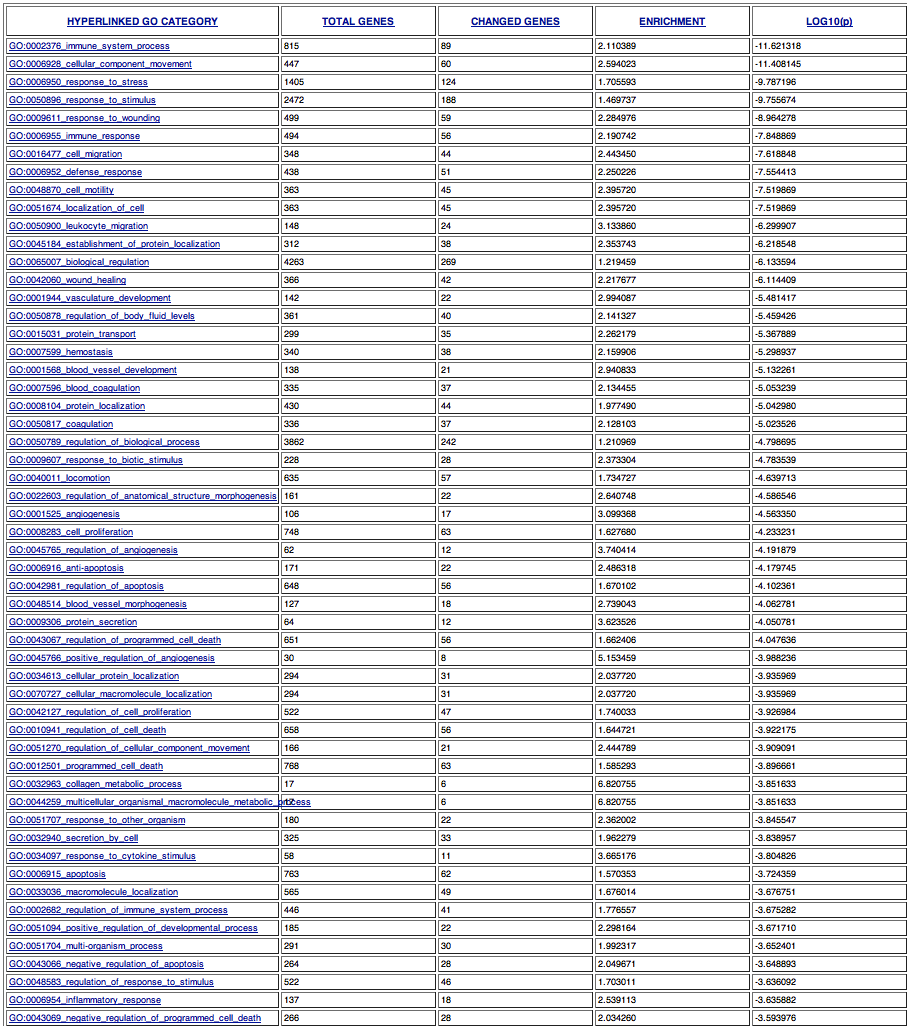


Green module


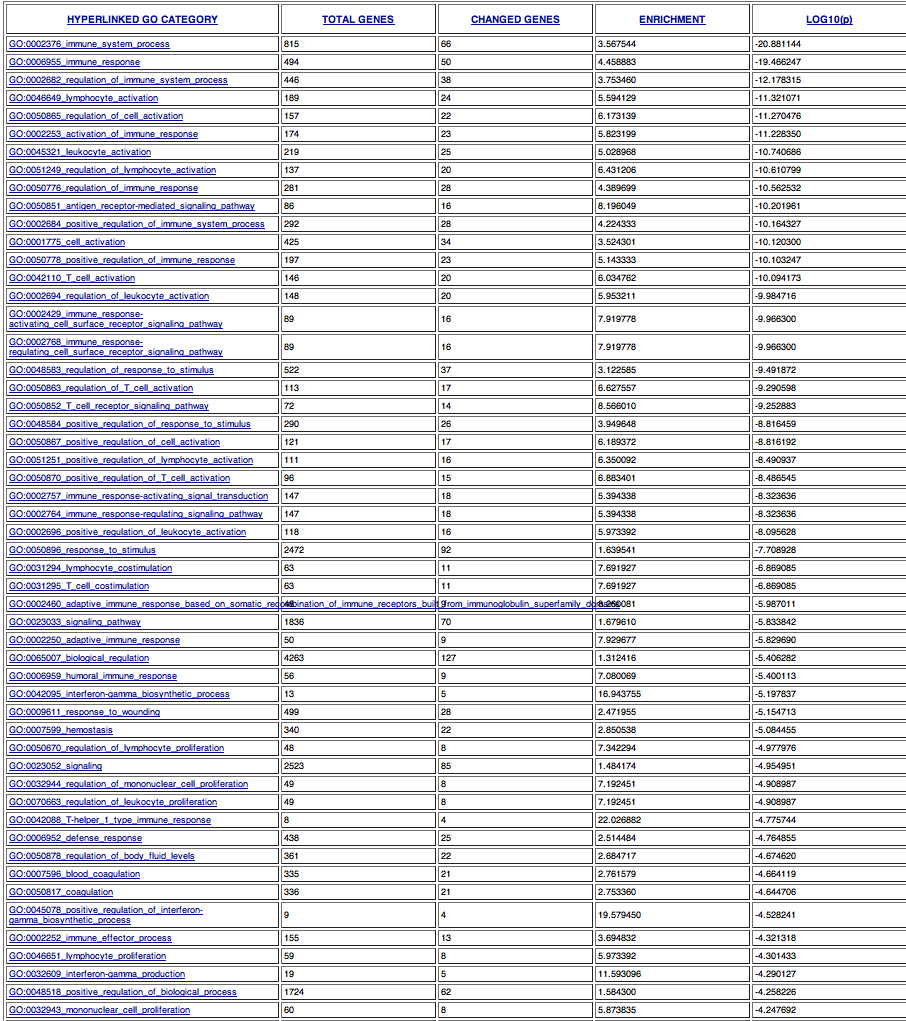


Red module


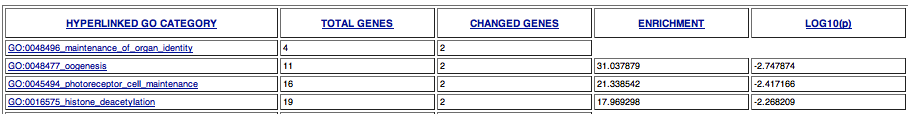


Turquoise module


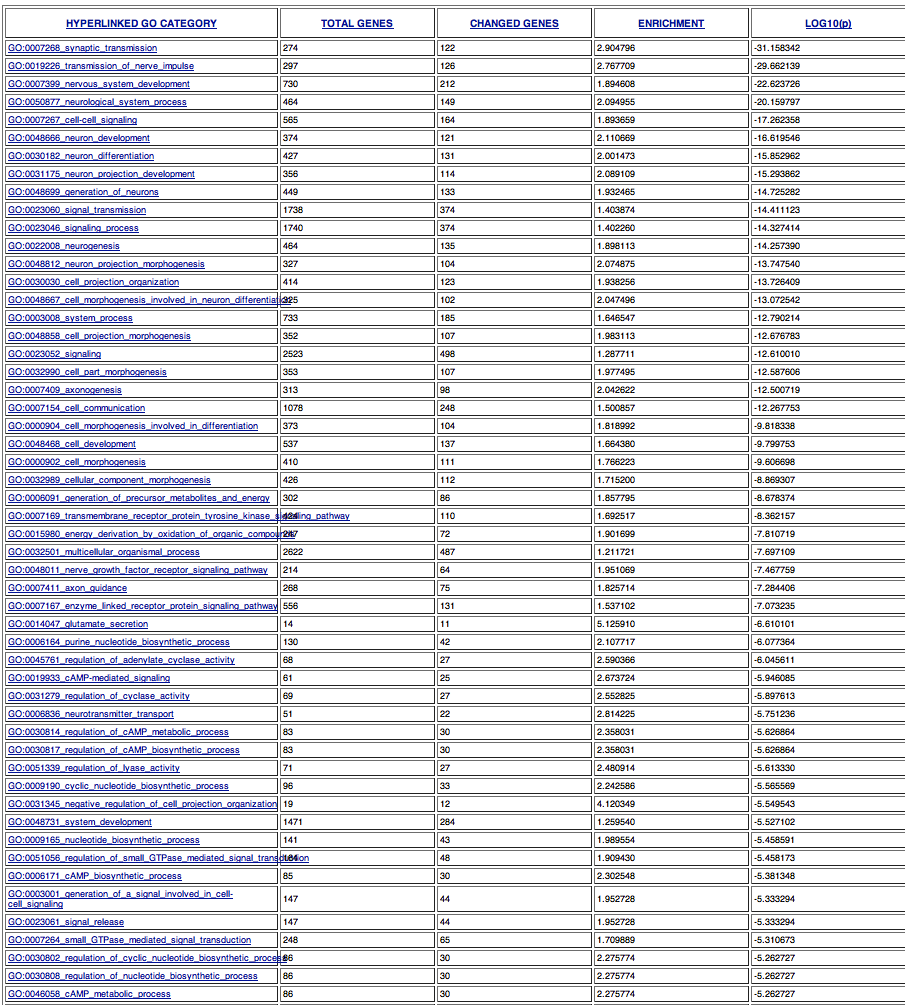


Yellow module


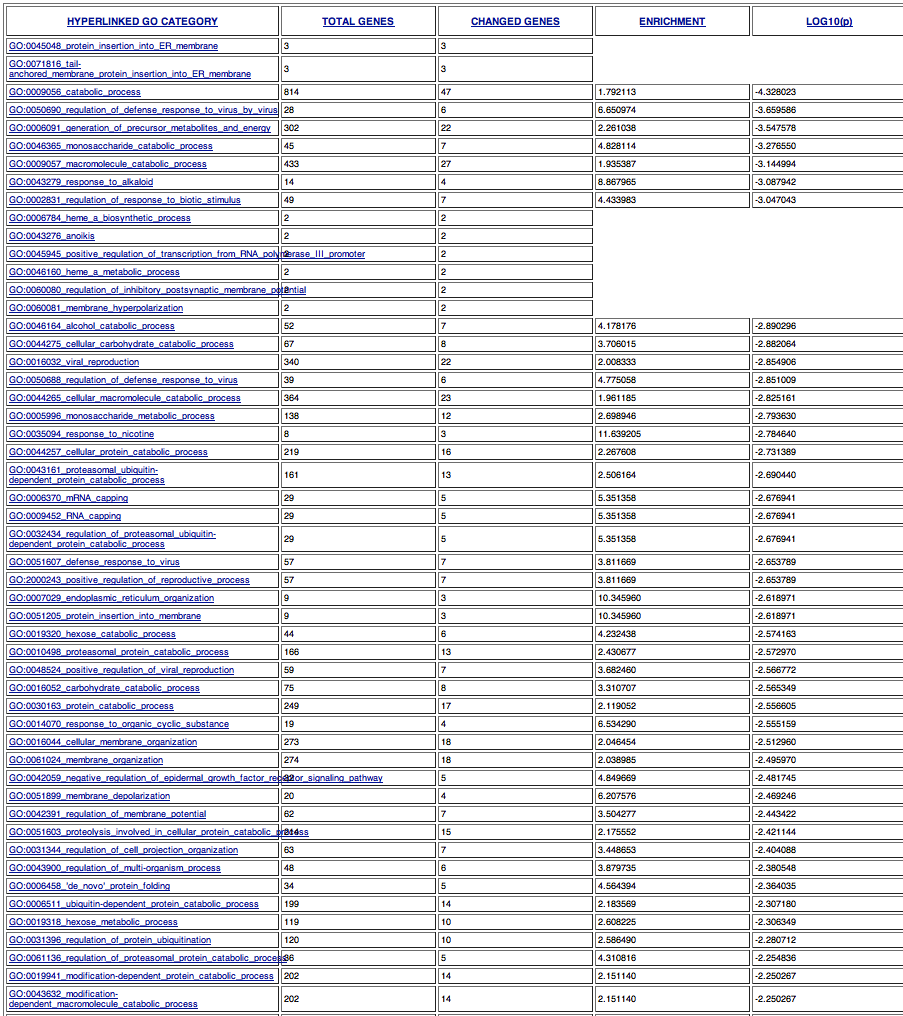

Supplement: Supplementary file 3 [file oncotarget-06-12094-s003.doc]
